# Pre-diagnosis Dietary One-Carbon Metabolism Micronutrients Consumption and Ovarian Cancer Survival: A Prospective Cohort Study

**DOI:** 10.3389/fnut.2022.873249

**Published:** 2022-04-15

**Authors:** He-Li Xu, Ting-Ting Gong, Fang-Hua Liu, Yi-Fan Wei, Hong-Yu Chen, Shi Yan, Yu-Hong Zhao, Song Gao, Yi-Sheng Jiao, Qi-Jun Wu

**Affiliations:** ^1^Department of Clinical Epidemiology, Shengjing Hospital of China Medical University, Shenyang, China; ^2^Clinical Research Center, Shengjing Hospital of China Medical University, Shenyang, China; ^3^Department of Obstetrics and Gynecology, Shengjing Hospital of China Medical University, Shenyang, China

**Keywords:** cohort, diet, one-carbon metabolism, ovarian cancer, survival

## Abstract

**Background and Aims:**

Epidemiological evidence on the relation between one-carbon metabolism (OCM) micronutrients intake and ovarian cancer (OC) survival are limited and conflicting. We evaluated the aforementioned associations in a prospective cohort-the Ovarian Cancer Follow-Up Study.

**Methods:**

A total of 635 newly diagnosed OC patients aged 18–79 y were enrolled in the present study. Dietary intake related to one-carbon metabolism, including methionine, vitamins B2, B3, B6, B9, B12, choline, and betaine, was assessed using a validated 111-item food frequency questionnaire. Deaths were ascertained until March 31, 2021, *via* medical records and active follow-up. Cox proportional hazards regression model was used to evaluate the hazard ratios (HRs) and 95% confidence intervals (CIs) for these aforementioned associations.

**Results:**

During a median follow-up of 37.2 months (interquartile: 24.7–50.2 months), 114 deaths were identified. We observed an improved survival with the highest compared with the lowest tertile of dietary vitamin B6 (HR = 0.52, 95%CI: 0.32–0.84, P-trend <0.05) and choline intake (HR = 0.50, 95%CI: 0.30–0.83, P-trend <0.05). No significant associations with OC survival were observed for dietary vitamins B2, B3, B9, B12, methionine, and betaine intake. We also observed a curvilinear association between vitamin B6 intake and OC survival (P non-linear <0.05).

**Conclusion:**

Our study suggests that pre-diagnosis higher intake of vitamin B6 and choline may improve OC survival. Further clarification of these associations is warranted.

## Introduction

Ovarian Cancer (OC) is one of the most common gynecologic cancers with a high mortality rate (1). In 2020, there were 3,13,959 new cases and 2,07,252 deaths of OC worldwide (2). The number of cases and deaths in China is 55,342 and 37,519 (3). Given the poor prognosis of this disease (4) and limited population-level strategies for early detection and long-term treatment success (5), knowledge of modifiable risk factors for prevention and improved prognosis is important.

During the past decades, there has been increasing epidemiological evidence of the relationship between inadequate intake of micronutrients and the appearance of tumor processes (6). The one-carbon metabolism (OCM) cycle is known to support multiple physiological processes essential for human development (7, 8), such as biosynthesis (purines and thymidine), amino acid homeostasis (glycine, serine, and methionine), epigenetic maintenance, and redox defense. Recent genomics and metabolomics approaches have also highlighted the distinctive aspects of OCM in cancer development and prognosis (9, 10). Therefore, micronutrients implicated in OCM —vitamins B2 (riboflavin), B3 (niacin), B6, B9 (folate), B12, methionine, choline, and betaine— deserve special attention.

One-carbon metabolism micronutrients are carriers or methyl-group donors (e.g., folates, choline, betaine, methionine) or cofactors of enzymes involved in the transfer reactions of these groups to DNA (namely vitamins B2, B6, and B12) (11). A dietary imbalance or deficiency in those micronutrients may disrupt DNA methylation or induce the disincorporation of nucleotide synthesis, which could lead to carcinogenesis (12–14). Although several observational studies have reported associations between individual OCM micronutrients and OC risk, the results of these studies are conflicting (14–24). For example, several case-control studies suggested null association between dietary folate intake and OC risk (15–19), whereas results from prospective studies suggested a modest inverse association (14, 20–22). Findings of methionine and vitamin B6 are also inconsistent. In the New England Case-Control Study, significant inverse associations between dietary methionine and vitamin B6 intake and OC risk were observed (23). However, the aforementioned associations were non-significant in the Nurses’ Health Study (14). Only one study previously investigated the relationship between dietary choline and betaine intake and the risk of OC and showed no association (24). Of note, recent, only two studies have investigated the relationship between individual OCM micronutrients and OC survival. For example, Dixon et al. (25) found no evidence that pre-diagnostic folate, vitamins B2, B6, and B12, methionine, betaine, or choline intake was associated with OC survival based on 1270 OC patients from Australia. However, Zhang et al. (26) observed that high folate intake was significantly associated with a lower risk of OC death based on 215 OC patients.

Notably, given these controversial results as well as the current lack of prospective evidence regarding the impact of dietary OCM micronutrients on OC survival, we present results from a prospective cohort, the Ovarian Cancer Follow-Up Study (OOPS), to clarify the associations of pre-diagnosis dietary consumption of OCM micronutrients with the survival of OC.

## Materials and Methods

### Study Design and Participants

The ovarian cancer follow-up study (OOPS) is a prospective longitudinal cohort study of patients newly diagnosed with OC to investigate the risk and prognostic factors for cancer-related outcomes. Complete details of the study design are available elsewhere (27–30). The OOPS was approved by the Institutional Review Board of the Ethics Committee of Shengjing Hospital of China Medical University, Shenyang, China, and informed consent was obtained from all patients.

**FIGURE 1:**
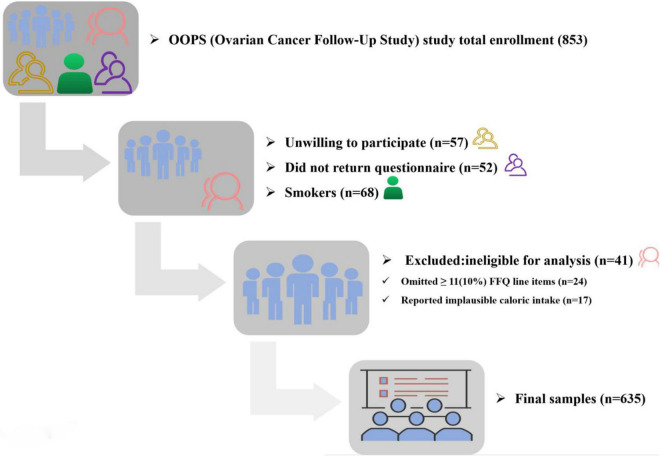
Flow of participants through study.

Between 2015 and 2020, 853 OC patients with 18–75 years of age were recruited at the Shengjing Hospital of China Medical University. Among them, 796 women (93%) consented to participate and 744 (87%) women returned the completed study questionnaire. For quality assurance of research, we excluded OC patients for: implausible caloric intake (<500 or >3500 calories per day; *n* = 17), 11 (10%) or more food items blank (*n* = 24). In addition, considering that smoking lowers serum folate and inhibits the one-carbon response (31, 32), smokers (*n* = 68) were likewise excluded. Finally, a total of 635 women were eligible for the analysis. Details are shown in the flow chart of the study participants (Figure 1).

### Data Collection

During the enrollment period, the participants were interviewed in-person by skilled interviewers with relevant medical knowledge. Information on socio-demographic characteristics including education, monthly household income, the levels of physical activity, cigarette smoking, medical and reproductive history, exogenous hormone use, anthropometric measures as well as alcohol consumption habits were gathered using lifestyle questionnaires as detailed elsewhere (27–30). Furthermore, clinical characteristics were extracted from the electronic medical records, including age at diagnosis, histological type (serious and non-serious), histopathologic grade (well, moderate, and poorly differentiated), International Federation of Gynecology and Obstetrics (FIGO) stage (I, II, III, IV, and unknown), residual lesions (none, <1, and ≥1 cm), and comorbidities (hypertension, coronary heart disease, diabetes, and so on) (yes and no).

### Dietary Exposure Assessment

Pre-diagnosis dietary intake was assessed at recruitment with a 111-item food frequency questionnaire (FFQ), which was previously validated (27, 28). The reproducibility coefficients (Spearman correlation coefficients and intraclass correlation coefficients) were above 0.5 for most food groups, and the correlation coefficients (Spearman correlation coefficients) were between 0.3 and 0.7 for most food groups between the FFQ and weighed dietary records. During the in-person interview, newly diagnosed OC patients reported their usual frequency of consumption of each food item in the 12 months before diagnosis. The frequency of intake ranged from almost none to twice a day or more. Nutrient contents of the food items were determined through a linkage of the FFQ responses to 2018 Chinese Food Composition Tables (33). Intakes of the following OCM micronutrients from food sources were available for analysis: vitamins B2 (riboflavin), B3 (niacin), B6, B9 (folate), B12, methionine, choline, and betaine. OCM micronutrients are thought to influence disease risk by donating methyl groups for methylation reactions (7, 34), we also calculated a “methyl-donor index” as a composite measure of dietary methyl intake by standardizing the nutrient intake levels on the log-scale [(nutrient value – mean)/standard deviation] then summed across all eight micronutrients, as described previously (35, 36). Intakes of OCM micronutrients from food sources were available for analysis. Each nutrient and methyl-donor index were adjusted for total energy intake based on the residual method (37).

### Cohort Follow-Up and Outcome Ascertainment

The OOPS participants were followed up until the occurrence of mortality from any cause or the last follow-up (March 31, 2021). Data on vital status were obtained by active follow-up and annual linkage to the Vital Statistics Unit in the Liaoning Centers for Disease Control and Prevention.

### Statistical Analysis

We calculated descriptive statistics of general and dietary characteristics. The Kaplan–Meier technique was used to plot crude survival curves and estimate the crude overall survival (OS) probabilities. Adjusted hazard ratios (HRs) and corresponding 95% confidence intervals (CIs) were derived from Cox proportional hazards regression model where the entry time was the date at which the OC patients enrolled in the OOPS and the exit time was the date when the participant died or was censored due to loss to follow-up or end of study follow-up on March 31, 2021, whenever occurred first. The proportional hazards assumption was tested through including an interaction term between dietary OCM nutrients and the logarithm of time, and no violations were found (all *p* > 0.05). The HR and 95%CI for each tertile were calculated using the first tertile as a reference. The linear trend of the association between dietary OCM nutrients intake and OC survival was assessed by assigning the median value of each tertile and treating it as continuous in a model. Continuous intakes were also calculated by per unit increase. We calculated age at diagnosis (continuous, years) and body mass index (BMI) (continuous, kg/m2)-adjusted HRs (Model 1). Model 2 was additionally adjusted for dietary changes (yes or no), alcohol drinking status (yes or no), education (junior secondary or below, senior high school/technical secondary school, and junior college/university or above), income (<5000, 5000–10000 or >10000 RMB), physical activity (continuous, MET-hours/day), menopausal status (yes or no), parity (≤1 or ≥2), multivitamin use (yes or no), multimineral use (yes or no), red meat intake (continuous, g/day), methyl-donor index (<5.22, 5.22–8.42, ≥8.42) and total energy. Model 3 was adjusted further for comorbidities (yes or no), FIGO stage (I-II, III-IV, or unknown), histological type (serious or non-serious), histopathologic grade (well, moderate, or poorly differentiated), residual lesions (none, <1, or ≥1 cm) to minimize the impact of clinical characteristics on survival. In our study, dietary change and parity were collected using a self-administered questionnaire. A restricted cubic spline model with three knots (i.e., 10, 50, and 90th percentiles) was also performed to test for non-linear relationships (38).

Stratified analyses were conducted by alcohol drinking (no and yes), age at diagnosis (<50 and ≥50 years), menopausal status (no and yes), FIGO stage (I-II and III-IV), residual lesions (no and yes), histological type (serious and non-serious), and BMI (<24 and ≥24 kg/m). Interactions were tested by using likelihood-ratio tests.

We conducted several sensitivity analyses to test the robustness of the primary findings. First, we restricted the study sample to participants among people who had not taken vitamin supplements. Second, we mutually adjusted for all of the dietary OCM nutrients to evaluate whether the associations were independent of each other (36). In addition, the data were analyzed in quartiles and compared with recommended intake (RI). Analyses were performed using SAS version 9.4 (SAS Institute, Cary, NC, United States). Two-sided *P*-values less than 0.05 were considered statistically significant.

## Results

During the median follow-up of 37.2 months (interquartile: 24.7–50.2 months), 114 deaths from all causes were recorded among all 635 patients. Table 1 summarizes the basic characteristics of OC patients. Later-stage disease and greater residual disease were statistically significantly associated with worse survival in this cohort (Supplementary Table 1).

**TABLE 1:** Baseline characteristics of ovarian cancer patients (*n* = 635).

Characteristics	All patients
No. of patients/deaths	635/114
Mean (SD) age at diagnosis (years)	53.76 (9.30)
Mean (SD) follow-up time (months)	32.33 (16.37)
Mean (SD) body mass index (kg/m^2^)	23.29 (3.61)
Mean (SD) physical activity (MET h/d)	15.65 (11.35)
Ever alcohol drinking	126 (19.84)
Ever tea drinking	192 (30.24)
Ever menopause	457 (71.97)
**Parity**	
≤1	473 (74.49)
≥2	162 (25.51)
**Educational level**	
Junior secondary or below	343 (54.02)
Senior high school/technical secondary school	127 (20.00)
Junior college/university or above	165 (25.98)
**Income per month (Yuan)**	
<5000	381 (60.00)
5000 to <10000	174 (27.40)
≥10000	80 (12.60)
Mean (SD) total energy intake (kcal/d)	1461.54 (555.10)
Mean (SD) carbohydrate intake (kcal/d)	913.00 (315.71)
Mean (SD) vegetable intake (kcal/d)	52.97 (29.78)
Mean (SD) fruit intake (kcal/d)	118.94 (92.57)
Mean (SD) meat intake (kcal/d)	72.02 (61.96)
Mean (SD) methionine intake (mg/d) *	1077.48 (256.85)
Mean (SD) vitamins B_2_ (riboflavin) intake (mg/d) *	0.89 (0.20)
Mean (SD) vitamins B_3_ (niacin) intake (mg/d) *	13.59 (2.50)
Mean (SD) vitamins B_6_ intake (mg/d) *	0.44 (0.12)
Mean (SD) vitamins B_9_ (folate) intake (μg/d) *	214.78 (73.88)
Mean (SD) vitamins B_12_ intake (μg/d) *	0.14 (0.20)
Mean (SD) choline intake (mg/d) *	279.17 (73.33)
Mean (SD) betaine intake (mg/d) *	57.16 (39.64)

MET, metabolic equivalents of task; SD, standard deviation. *Energy adjustment by residual method. Values are numbers (percentages) unless stated otherwise.

**FIGURE 2:**
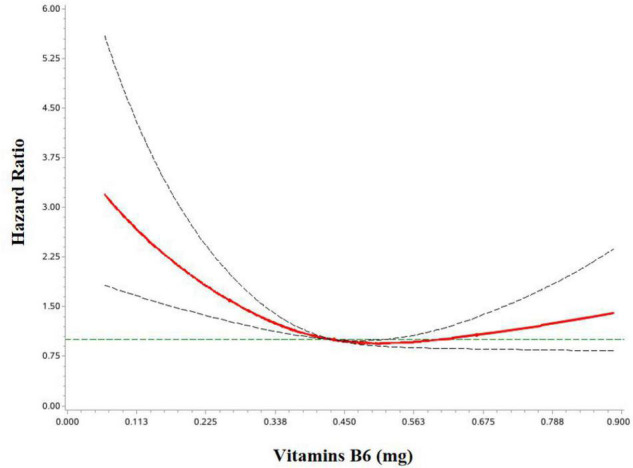
HR and 95%CIs of overall survival among OC patients by vitamin B6. The association was adjusted for age at diagnosis, body mass index, total energy, alcohol drinking, diet change, education, income, physical activity, menopausal status, parity, multivitamin use, multimineral use, red meat, methyl-donor index, comorbidities, FIGO stage, histological type, histopathologic grade, and residual lesions.

Multivariable-adjusted HRs and 95%CIs for associations between dietary intakes of OCM micronutrients and OC survival are shown in Table 2. Higher dietary vitamin B6 intake was associated with lower mortality of OC (HR Tertile 3 vs. Tertile 1 = 0.52; 95%CI = 0.32–0.84; P trend <0.05). Additionally, dietary choline intake was associated with a decreased OC survival (HR Tertile 3 vs. Tertile 1 = 0.50; 95% CI = 0.30–0.83; P trend <0.05) (Supplementary Figure 1). However, we failed to observe significant associations for OC mortality with the intake of vitamins B2, B3, B9, B12, methionine, and betaine. Of note, we observed a curvilinear association between vitamin B6 intake and OC survival (P non-linear <0.05) (Figure 2 and Supplementary Figure 2).

**TABLE 2:** Adjusted hazard ratio (HR) and 95% confidence intervals (CIs) for the association between dietary one-carbon metabolism micronutrients intake and total mortality of ovarian cancer (*n* = 635)*.

Characteristics	Tertiles of energy-adjusted intake **			*P* trend ^†^	Continuous ^‡^
	I	II	III		
Methionine (Range, mg/d)	<979.81	979.81–1103.40	≥1103.40		
Deaths, N (% of total deaths)	38 (33.33)	44 (38.60)	32 (28.07)		
Model 1	1.00 (Ref)	1.07 (0.69–1.66)	0.75 (0.46–1.20)	0.19	0.92 (0.79–1.06)
Model 2	1.00 (Ref)	1.15 (0.73–1.81)	0.84 (0.52–1.38)	0.44	0.93 (0.80–1.07)
Model 3	1.00 (Ref)	1.23 (0.77–1.96)	0.85 (0.51–1.40)	0.44	0.93 (0.80–1.07)
Vitamins B_2_ (riboflavin) (Range, mg/d)	<0.82	0.82–0.96	≥0.96		
Deaths, N (% of total deaths)	40 (35.09)	38 (33.33)	36 (31.58)		
Model 1	1.00 (Ref)	0.93 (0.60–1.45)	0.86 (0.54–1.35)	0.50	0.90 (0.73–1.11)
Model 2	1.00 (Ref)	0.88 (0.56–1.40)	0.88 (0.55–1.40)	0.60	0.91 (0.74–1.11)
Model 3	1.00 (Ref)	0.96 (0.60–1.56)	0.93 (0.58–1.51)	0.71	0.93 (0.76–1.14)
Vitamins B_3_ (niacin) (Range, mg/d)	<12.52	12.52–14.85	≥14.85		
Deaths, N (% of total deaths)	40 (35.09)	40 (35.09)	34 (29.82)		
Model 1	1.00 (Ref)	1.04 (0.67–1.62)	0.77 (0.48–1.22)	0.31	0.89 (0.69–1.15)
Model 2	1.00 (Ref)	0.96 (0.61–1.51)	0.70 (0.42–1.15)	0.18	0.88 (0.67–1.17)
Model 3	1.00 (Ref)	0.99 (0.63–1.56)	0.77 (0.46–1.28)	0.35	0.93 (0.70–1.24)
Vitamins B_6_ (Range, mg/d)	<0.39	0.39–0.48	≥0.48		
Deaths, N (% of total deaths)	48 (42.11)	34 (29.82)	32 (28.07)		
Model 1	1.00 (Ref)	0.59 (0.38–0.92)	0.61 (0.39–0.95)	<0.05	0.75 (0.58–0.97)
Model 2	1.00 (Ref)	0.52 (0.33–0.81)	0.54 (0.34–0.86)	<0.05	0.73 (0.57–0.95)
Model 3	1.00 (Ref)	0.48 (0.30–0.76)	0.52 (0.32–0.84)	<0.05	0.70 (0.53–0.92)
Vitamins B_9_ (folate) (Range, μg/d)	<184.77	184.77–233.25	≥233.25		
Deaths, N (% of total deaths)	39 (34.21)	40 (35.09)	35 (30.70)		
Model 1	1.00 (Ref)	0.97 (0.62–1.50)	0.78 (0.49–1.23)	0.26	0.85 (0.70–1.04)
Model 2	1.00 (Ref)	0.87 (0.56–1.38)	0.72 (0.45–1.12)	0.18	0.82 (0.67–1.00)
Model 3	1.00 (Ref)	0.78 (0.49–1.25)	0.75 (0.47–1.20)	0.24	0.81 (0.65–1.00)
Vitamins B_12_ (Range, μg/d)	<0.05	0.05–0.14	≥0.14		
Deaths, N (% of total deaths)	34 (29.82)	42 (36.85)	38 (33.33)		
Model 1	1.00 (Ref)	1.23 (0.78–1.93)	1.04 (0.65–1.65)	0.99	1.02 (0.91–1.16)
Model 2	1.00 (Ref)	1.34 (0.81–2.19)	1.03 (0.64–1.66)	0.83	1.00 (0.89–1.14)
Model 3	1.00 (Ref)	1.42 (0.86–2.35)	1.02 (0.63–1.65)	0.75	1.02 (0.90–1.17)
Choline (Range, mg/d)	<245.60	245.60–310.02	≥310.02		
Deaths, N (% of total deaths)	46 (40.35)	41 (35.96)	27 (23.69)		
Model 1	1.00 (Ref)	0.82 (0.54–1.26)	0.54 (0.33–0.87)	<0.05	0.78 (0.61–0.98)
Model 2	1.00 (Ref)	0.67 (0.43–1.04)	0.51 (0.31–0.84)	<0.05	0.76 (0.60–0.97)
Model 3	1.00 (Ref)	0.67 (0.42–1.06)	0.50 (0.30–0.83)	<0.05	0.79 (0.61–1.01)
Betaine (Range, mg/d)	<41.35	41.35–61.55	≥61.55		
Deaths, N (% of total deaths)	34 (29.82)	40 (35.09)	40 (35.09)		
Model 1	1.00 (Ref)	1.09 (0.69–1.73)	1.12 (0.71–1.78)	0.64	1.01 (0.86–1.20)
Model 2	1.00 (Ref)	1.22 (0.75–1.97)	1.21 (0.76–1.94)	0.47	1.01 (0.87–1.20)
Model 3	1.00 (Ref)	1.15 (0.70–1.86)	1.12 (0.70–1.80)	0.69	0.98 (0.83–1.16)

*CI, confidence interval; HR, hazard ratio; Ref, reference.*

*HR and 95% CI were calculated with the use of the Cox proportional hazards regression model.

**Adjusted for energy by the residual method.

*†Test for trend based on variables containing the median value for each tertile.*

*‡ Continuous intakes were calculated by per unit increase.*

*Model 1 adjusted for age at diagnosis and body mass index.*

*Model 2 adjusted for age at diagnosis, total energy, body mass index, alcohol drinking, diet change, education, income, physical activity, menopausal status, parity, multivitamin use, multimineral use, red meat, and methyl-donor index.*

*Model 3 adjusted for age at diagnosis, total energy, body mass index, alcohol drinking, diet change, education, income, physical activity, menopausal status, parity, multivitamin use, multimineral use, red meat, methyl-donor index, comorbidities, FIGO stage, histological type, histopathologic grade, and residual lesions.*

No significant interactions were found in the subgroup analyses stratified by demographic and clinical characteristics (Table 3). The direction of these results was mainly consistent with the main findings but not all of them showed statistical significance. The inverse associations between vitamin B6 and choline intake and OC mortality seemed slightly stronger in patients with an age greater than 50 years, menopausal status, serious patients, and no residual lesion patients (Supplementary Tables 2–4). In addition, the inverse association between vitamin B6 intake and OC mortality was slightly stronger in OC patients with BMI ≥24, alcohol drinkers, and stage III–IV, while the protective effect of choline was stronger in those with BMI <24, non-drinkers and stage I-II patients.

**TABLE 3:** Subgroup analyses for adjusted hazard ratio (HR) and 95% confidence intervals (CIs) for the association between dietary vitamin B_6_ and choline intake and total mortality of ovarian cancer (*n* = 635).

Characteristics	Tertiles of energy-adjusted intake*							
	Vitamins B_6_				Choline			
	I	II	III	*P*_*interaction*_ **	I	II	III	*P*_*interaction*_ **
**Age at diagnosis (years)**				0.32				0.75
≤50	1.00 (Ref)	0.31 (0.12–0.83)	0.84 (0.34–2.08)		1.00 (Ref)	0.67 (0.27–1.65)	0.81 (0.31–2.15)	
>50	1.00 (Ref)	0.49 (0.27–0.89)	0.39 (0.21–0.74)		1.00 (Ref)	0.55 (0.31–0.98)	0.40 (0.21–0.75)	
**Menopausal status**				0.45				0.38
No	1.00 (Ref)	0.17 (0.05–0.55)	0.55 (0.18–1.71)		1.00 (Ref)	0.65 (0.24–1.74)	0.49 (0.13–1.86)	
Yes	1.00 (Ref)	0.56 (0.32–1.00)	0.51 (0.29–0.91)		1.00 (Ref)	0.57 (0.33–1.00)	0.52 (0.29–0.95)	
**Body mass index (kg/m^2^)**				0.83				0.09
<24	1.00 (Ref)	0.77 (0.40–1.45)	0.74 (0.38–1.42)		1.00 (Ref)	0.50 (0.26–0.96)	0.59 (0.31–1.15)	
≥24	1.00 (Ref)	0.19 (0.09–0.42)	0.25 (0.10–0.63)		1.00 (Ref)	0.81 (0.38–0.1.72)	0.42 (0.17–1.05)	
**Alcohol drinking**				0.42				0.80
No	1.00 (Ref)	0.54 (0.31–0.93)	0.60 (0.35–1.03)		1.00 (Ref)	0.62 (0.36–1.07)	0.44 (0.25–0.79)	
Yes	1.00 (Ref)	0.15 (0.04–0.54)	0.10 (0.02–0.46)		1.00 (Ref)	0.90 (0.29–2.77)	0.69 (0.17–2.85)	
**Histological type**				0.75				0.99
Serous	1.00 (Ref)	0.45 (0.26–0.79)	0.46 (0.25–0.84)		1.00 (Ref)	0.71 (0.40–1.25)	0.51 (0.27–0.95)	
Non-serous	1.00 (Ref)	0.45 (0.16–1.24)	0.59 (0.23–1.56)		1.00 (Ref)	0.47 (0.16–1.35)	0.40 (0.13–1.23)	
**FIGO stage**				0.89				0.96
I–II	1.00 (Ref)	0.53 (0.22–1.28)	0.47 (0.19–1.14)		1.00 (Ref)	0.23 (0.09–0.60)	0.21 (0.08–0.56)	
III–IV	1.00 (Ref)	0.35 (0.20–0.64)	0.47 (0.25–0.88)		1.00 (Ref)	0.73 (0.39–1.37)	0.52 (0.27–1.03)	
**Residual lesions**				0.48				0.25
No	1.00 (Ref)	0.52 (0.29–0.93)	0.48 (0.26–0.89)		1.00 (Ref)	0.50 (0.29–0.89)	0.43 (0.23–0.82)	
Yes	1.00 (Ref)	0.28 (0.11–0.73)	0.55 (0.22–1.35)		1.00 (Ref)	1.21 (0.52–2.82)	0.79 (0.29–2.15)	

*CI, confidence interval; HR, hazard ratio; Ref, reference.*

*Adjusted for energy by the residual method.

**Test for interaction based on strata and dietary vitamin B6 and Choline intake. HR and 95% CI were calculated with the use of the Cox proportional hazards regression model with adjustment for age at diagnosis, body mass index, total energy, alcohol drinking, diet change, education, income, physical activity, menopausal status, parity, multivitamin use, multimineral use, red meat, methyl-donor index, comorbidities, FIGO stage, histological type, histopathologic grade, and residual lesions.

In sensitivity analysis that excluded women who had taken vitamin supplements, the results were equivalent to the original analysis (Supplementary Table 5). Furthermore, the association for dietary vitamin B6 and choline intake remained significant in the most elaborate model that included all eight micronutrients (Supplementary Table 6). We have also carried out analyses in quartile comparison and the results are consistent with those of tertiles (Supplementary Table 7). Vitamin B2, B3, B9, and choline did not show an association with OC survival when grouped according to RI (Supplementary Table 8).

## Discussion

The present study is one of the limited studies evaluating the role of pre-diagnosis dietary OCM nutrients in the survival of OC. This paper highlights the inverse, statistically significant relationship between pre-diagnostic dietary vitamin B6 and choline intake levels and OC survival. Null associations were observed for vitamins B2, B3, B9, B12, methionine, and betaine. Further, the relationship between vitamin B6 intake and OC survival was curvilinear.

To date, only two observational studies (25, 26) examined the association between pre-diagnosis OCM nutrients and OC survival. One study suggested null associations between OCM nutrients intake and OC survival, another study indicated that folate intake was significantly associated with a lower risk of OC death. However, our findings were partly inconsistent with both of them. Possible explanations for the discrepancy might be attributed to the different demographic and clinical characteristics of OC patients, FFQ measurements, dietary habits, sample size and follow-up periods. For example, compared with these two previous studies, we included a moderate sample size (635 vs. 1270 and 215) and had a shorter follow-up period (3.1 years vs. 10 and 4.02 years). In addition, more advanced FIGO stage III-IV (71.02% vs. 48.35%) and diagnostic age >50 (81.8% vs. 63.46%) were included in the study by Dixon et al. than us (25). Furthermore, Zhang et al. failed to adjust for key confounding factors including FIGO stage, tumor grade, and presence of residual disease (26). Dietary habits in different countries and different regions of the same country may also provide insights into these contradictions.

Our study suggested that higher intake of vitamin B6 and choline was associated with better OC survival among post-menopausal women. Our previous study suggested that pre-diagnosis cruciferous vegetables intake was only associated better survival of OC in postmenopausal patients when stratified by menopausal status (28). These results are consistent with previous studies that have shown a stronger effect of some dietary nutrients (such as alpha-carotene, h-cryptoxanthin) in reducing OC incidence in postmenopausal rather than in premenopausal women (39, 40). Studies have demonstrated that dietary nutrients intake can alter circulating levels of estrogen and other sex hormones (41). The possible mechanism might partially lie in that vitamin B6 and choline affect the ovarian synthesis of sex hormones or the alteration of other menstrual cycle characteristics (42). Vitamin B6 and choline may be effective only at low sex hormone concentrations which was shown in postmenopausal women. Further studies are recommended to assess dietary OCM nutrients intake and OC survival separately in pre- and post-menopausal women.

A protective effect of vitamin B6 on OC survival is biologically plausible given vitamin B6’s role as a cofactor for enzymes involved in the DNA synthesis and methylation pathways of OCM (43). A diet low in vitamin B6 results in a decreased production of the methyl donor, methylene-tetrahydrofolate (44) and eventually leads to chromosome breaks and thus involvement in tumor progression (45). In addition, laboratory studies have demonstrated that vitamin B6 is effective at scavenging free radicals which if not properly controlled can promote carcinogenesis (19), so vitamin B6 may influence OC through its antioxidant properties. The mean dietary intake of choline in our study was 279.17 mg/day, which is relatively lower than its recommended intake (RI = 400 mg/d). Choline is a methyl-group donor involved in OCM cycle, abnormal choline metabolism is emerging as a metabolic hallmark that is associated with oncogenesis and tumor progression (46, 47). Humans ingest approximately 50 mmol of methyl groups per day, and 60% of them are derived from choline. Animals fed diets deficient in methyl donors (choline) have hypomethylated DNA (48). These changes occur not only in global methylation, but also in the methylation of specific genes (49, 50), which can easily be influenced by changes in human diet. This proven scientific insight promises to enhance our understanding of how choline affects the prognosis of OC.

In our study, we found that the mean dietary intake of vitamin B2, B6, B9, B12, and choline was less than the Dietary Nutrient Reference Intakes for Chinese Residents. This phenomenon is also observed in several previous studies (19, 51–53). For example, the mean dietary vitamin B9 intake of OC patients were below the recommended intake (346 ug/d < RI = 400 ug/d) in a case-control study from the United States (19). A hospital-based case-control study from Hong Kong, China also showed that the mean dietary vitamin B6 (0.79 ug/d < RI = 1.6 ug/d) and B9 (207.4 ug/d < RI = 400 ug/d) intakes were relatively lower in breast cancer cases (52). However, these phenomena were not common in the general population (22, 54).

Currently little is known about the possible relation between vitamin-B12 and cancer risk. However, since vitamin-B12 has a key role in one-carbon metabolism and cells require one-carbon units for DNA synthesis, methylation as well as redox and reductive metabolism, vitamin-B12 may influence pathways enhancing the proliferation of cancer cells (55). However, we found a non-significant association between dietary vitamin B12 intake and OC survival. This might be partly attributed to the low intake of dietary vitamin B12 in OC patients (mean value is 0.14 μg/d). According to the ChineseDRIs, the value should be 2.4 ug/d. In addition, compared to several previous studies (25, 51, 53, 56), dietary vitamin B12 intake was relatively lower in our study. This difference might be attributed to the different study participants and dietary habit. Furthermore, coupled with the difficulty of accurately measuring vitamin B12 (57), this may lead to greater changes and weaken these links.

Strengths of our study include data that were prospectively collected in high-quality population-based registers, reducing bias in ascertainment of the exposure and outcomes. Another strength of this study is one of the limited studies to assess the association between pre-diagnosis OCM nutrients intake and OC survival. Also, the collection of numerous clinical and lifestyle covariates related to OC survival allowed for statistical adjustment of these factors to limit potential confounding. A reproducible and validated FFQ also enabled us to achieve a comprehensive dietary intake assessment of OCM nutrients.

Potential limitations of our study should also be considered. First, information on dietary intake and other covariates were self-reported, and therefore, non-differential misclassification of these variables resulting from recall and reporting biases was possible. However, we used a highly reproducible validated FFQ and selected highly trained and skilled researchers to collect information. Second, the current study was the single assessment of diet, which eliminated the possibility to examine dietary changes during follow-up. Whereas, we have adjusted for dietary change as a confounder. Third, because regular use of any type of supplement was rare in our cohort (<10%), we were only able to assess food sources of nutrients. Meanwhile, the finding of the sensitivity analysis in the population not taking B vitamin supplements was consistent with the main analysis. Fourth, we did not determine the internal levels of these nutrients in the body, and the relatively less precise assessment by the FFQ may have attenuated any associations. Lastly, we could not evaluate the association between OCM micronutrients and OC specific mortality because the data for the cause of death were not available. Thus, our findings should be interpreted cautiously and need to be confirmed by future studies.

In summary, this prospective cohort study demonstrated inverse associations between pre-diagnostic dietary vitamin B6 and choline and OC survival. Future large prospective cohorts should validate our findings and would improve our understanding of the role of OCM micronutrients intake in the prognosis of OC.

## Data Availability Statement

The raw data supporting the conclusions of this article will be made available by the authors, without undue reservation.

## Ethics Statement

The studies involving human participants were reviewed and approved by The Institutional Review Board of the Ethics Committee of Shengjing Hospital of China Medical University, Shenyang, China. The patients/participants provided their written informed consent to participate in this study.

## Author Contributions

H-LX, T-TG, and Q-JW conceived the study. T-TG, Y-HZ, SG, Y-SJ, and Q-JW contributed to the design. H-LX, T-TG, SY, and SG collected the data. H-LX, F-HL, Y-FW, SY, and Q-JW cleaned the data and checked the discrepancy. H-LX, F-HL, Y-FW, and H-YC analyzed the data. H-LX, T-TG, F-HL, Y-FW, H-YC, and Q-JW interpreted the data. All authors interpreted the data, read the manuscript, and approved the final vision.

## Conflict of Interest

The authors declare that the research was conducted in the absence of any commercial or financial relationships that could be construed as a potential conflict of interest.

## Publisher’s Note

All claims expressed in this article are solely those of the authors and do not necessarily represent those of their affiliated organizations, or those of the publisher, the editors and the reviewers. Any product that may be evaluated in this article, or claim that may be made by its manufacturer, is not guaranteed or endorsed by the publisher.
