# Supplementary material for: Pre-diagnosis Dietary One-Carbon Metabolism Micronutrients Consumption and Ovarian Cancer Survival: A Prospective Cohort Study
Source: Front Nutr. 2022 Apr 15;9:873249. doi: 10.3389/fnut.2022.873249 (PMC9053828; doi:10.3389/fnut.2022.873249)
Supplement: Supplementary file 1 [file Data_Sheet_1.pdf]

Supplementary Table 1. Selected clinical characteristics and associations with overall survival among ovarian cancer patients (N=635)

Characteristics	No. of deaths/total (%)	Adjusted HR * (95%CI)
**Age at diagnosis**		
≤ 50 years	39/232 (6.14)	1.00 (ref)
> 50 years	75/403 (11.81)	1.26 (0.85-1.87)
**Histological type**		
Serous	80/430 (12.60)	1.00 (ref)
Non-serous	34/205 (5.35)	1.57 (0.99-2.50)
**Histopathologic grade**		
Well differentiated	4/49 (0.63)	1.00 (ref)
Moderately differentiated	7/45 (1.10)	1.24 (0.36-4.31)
Poorly differentiated	103/541 (16.22)	1.78 (0.64-4.99)
**FIGO stage**		
I-II	37/306 (5.83)	1.00 (ref)
III-IV	77/307 (12.13)	2.44 (1.55-3.83)
**Residual lesions**		
No	74/499 (11.65)	1.00 (ref)
<1 cm	28/99 (4.41)	1.66 (1.05-2.64)
≥1 cm	12/37 (1.89)	1.94 (1.03-3.65)
**Comorbidities**		
No	64/355 (10.08)	1.00 (ref)
Yes	50/280 (7.87)	0.96 (0.66-1.41)

CI, confidence interval; FIGO, the International Federation of Gynecology and Obstetrics; HR, hazard ratio; Ref, reference.

* Mutually adjusted for all other variables listed in the table.

Supplementary Table 2. Subgroup analyses for adjusted hazard ratio (HR) and 95% confidence intervals (CIs) for the association between dietary vitamin B_2 and B_3 intake and total mortality of ovarian cancer (n=635)

Characteristics	Tertiles of energy-adjusted intake *							
	vitamin B_2				vitamin B_3			
	I	II	III	$P_{interaction}$ **	I	II	III	$P_{interaction}$ **
**Age at diagnosis (years)**				0.34				0.65
≤ 50	1.00 (Ref)	1.14 (0.46-2.82)	2.05 (0.81-5.23)		1.00 (Ref)	0.72 (0.30-1.74)	0.75 (0.20-1.61)	
> 50	1.00 (Ref)	0.75 (0.41-1.37)	0.76 (0.42-1.37)		1.00 (Ref)	0.95 (0.52-1.73)	0.82 (0.44-1.53)	
**Menopausal status**				0.43				0.51
No	1.00 (Ref)	1.06 (0.36-3.19)	1.65 (0.51-5.32)		1.00 (Ref)	1.30 (0.45-3.77)	0.61 (0.17-2.21)	
Yes	1.00 (Ref)	0.78 (0.43-1.41)	0.84 (0.48-1.47)		1.00 (Ref)	0.82 (0.47-1.44)	0.79 (0.44-1.44)	
**Body mass index (kg/m^2)**				0.23				< 0.05
< 24	1.00 (Ref)	1.18 (0.62-2.25)	1.01 (0.52-1.98)		1.00 (Ref)	0.77 (0.42-1.42)	0.64 (0.31-1.30)	
≥ 24	1.00 (Ref)	0.74 (0.33-1.66)	0.87 (0.39-1.92)		1.00 (Ref)	1.97 (0.84-4.63)	0.81 (0.33-1.97)	
**Alcohol drinking**				0.43				0.09
No	1.00 (Ref)	0.83 (0.47-1.44)	0.75 (0.43-1.32)		1.00 (Ref)	0.73 (0.43-1.24)	0.66 (0.37-1.18)	
Yes	1.00 (Ref)	1.16 (0.34-3.97)	1.86 (0.60-5.75)		1.00 (Ref)	2.38 (0.78-7.29)	0.99 (0.27-3.65)	
**Histological type**				0.75				0.05
Serous	1.00 (Ref)	1.15 (0.66-2.04)	0.96 (0.54-1.72)		1.00 (Ref)	1.39 (0.78-2.47)	1.02 (0.54-1.90)	
Non-serous	1.00 (Ref)	0.50 (0.17-1.52)	0.65 (0.22-1.90)		1.00 (Ref)	0.60 (0.25-1.47)	0.25 (0.08-0.85)	
**FIGO stage**				0.78				0.79
I-II	1.00 (Ref)	0.42 (0.17-1.05)	0.50 (0.21-1.17)		1.00 (Ref)	0.50 (0.20-1.25)	0.53 (0.21-1.34)	

III-IV	1.00 (Ref)	1.17 (0.63-2.17)	1.14 (0.61-2.14)		1.00 (Ref)	1.26 (0.72-2.21)	0.77 (0.39-1.52)	
**Residual lesions**				0.05				0.41
No	1.00 (Ref)	0.63 (0.36-1.13)	0.63 (0.35-1.15)		1.00 (Ref)	1.12 (0.64-1.95)	0.65 (0.34-1.26)	
Yes	1.00 (Ref)	2.45 (0.97-6.20)	2.48 (0.95-6.50)		1.00 (Ref)	0.90 (0.36-2.25)	1.34 (0.53-3.42)	

CI, confidence interval; HR, hazard ratio; Ref, reference.

*****Adjusted for energy by the residual method.

******Test for interaction based on strata and dietary vitamin B_2 and B_3 intake.

HR and 95% CI were calculated with the use of the Cox proportional hazards regression model with adjustment for age at diagnosis, body mass index, total energy, alcohol drinking, diet change, education, income, physical activity, menopausal status, parity, multivitamin use, multimineral use, red meat, methyl-donor index, comorbidities, FIGO stage, histological type, histopathologic grade, and residual lesions.

Supplementary Table 3. Subgroup analyses for adjusted hazard ratio (HR) and 95% confidence intervals (CIs) for the association between dietary vitamin B_9 and B_{12} intake and total mortality of ovarian cancer (n=635)

Characteristics	Tertiles of energy-adjusted intake [*]							
	vitamin B_9				vitamin B_{12}			
	I	II	III	$P_{interaction}$ [**]	I	II	III	$P_{interaction}$ [**]
**Age at diagnosis (years)**				0.78				0.14
≤ 50	1.00 (Ref)	0.84 (0.32-2.19)	1.08 (0.43-2.67)		1.00 (Ref)	2.38 (0.89-6.38)	1.17 (0.45-3.08)	
> 50	1.00 (Ref)	0.78 (0.45-1.36)	0.65 (0.37-1.17)		1.00 (Ref)	1.06 (0.57-1.96)	0.95 (0.52-1.72)	
**Menopausal status**				0.89				0.30
No	1.00 (Ref)	0.61 (0.20-1.83)	0.68 (0.21-2.19)		1.00 (Ref)	2.13 (0.65-6.92)	1.78 (0.59-5.34)	
Yes	1.00 (Ref)	0.77 (0.44-1.32)	0.79 (0.45-1.36)		1.00 (Ref)	1.30 (0.73-2.32)	0.90 (0.51-1.59)	
**Body mass index (kg/m^2)**				0.80				0.25
< 24	1.00 (Ref)	0.97 (0.51-1.84)	0.78 (0.41-1.51)		1.00 (Ref)	1.38 (0.71-2.69)	0.94 (0.49-1.83)	
≥ 24	1.00 (Ref)	0.59 (0.27-1.30)	0.66 (0.30-1.45)		1.00 (Ref)	1.28 (0.56-2.94)	1.11 (0.52-2.38)	
**Alcohol drinking**				0.60				0.63
No	1.00 (Ref)	0.72 (0.42-1.24)	0.74 (0.43-1.26)		1.00 (Ref)	1.54 (0.85-2.80)	1.13 (0.65-1.98)	
Yes	1.00 (Ref)	0.83 (0.29-2.38)	0.49 (0.14-1.70)		1.00 (Ref)	2.95 (0.81-10.83)	0.67 (0.18-2.49)	
**Histological type**				0.56				0.43
Serous	1.00 (Ref)	0.84 (0.48-1.49)	0.89 (0.50-1.59)		1.00 (Ref)	1.40 (0.77-2.52)	0.88 (0.50-1.55)	
Non-serous	1.00 (Ref)	0.59 (0.23-1.47)	0.52 (0.20-1.33)		1.00 (Ref)	1.99 (0.64-6.24)	1.60 (0.53-4.84)	
**FIGO stage**				0.73				0.70
I-II	1.00 (Ref)	0.79 (0.34-1.85)	0.48 (0.20-1.14)		1.00 (Ref)	0.82 (0.31-2.16)	1.15 (0.47-2.80)	

III-IV	1.00 (Ref)	0.73 (0.40-1.33)	0.94 (0.53-1.68)		1.00 (Ref)	2.20 (1.17-4.14)	1.16 (0.64-2.10)	
**Residual lesions**				0.77				0.62
No	1.00 (Ref)	0.85 (0.48-1.51)	0.77 (0.43-1.37)		1.00 (Ref)	1.11 (0.60-2.05)	0.88 (0.49-1.59)	
Yes	1.00 (Ref)	0.65 (0.28-1.50)	0.80 (0.32-2.00)		1.00 (Ref)	1.84 (0.73-4.60)	1.44 (0.59-3.51)	

CI, confidence interval; HR, hazard ratio; Ref, reference.

*Adjusted for energy by the residual method.

** Test for interaction based on strata and dietary vitamin B_9 and B_{12} intake.

HR and 95% CI were calculated with the use of the Cox proportional hazards regression model with adjustment for age at diagnosis, body mass index, total energy, alcohol drinking, diet change, education, income, physical activity, menopausal status, parity, multivitamin use, multimineral use, red meat, methyl-donor index, comorbidities, FIGO stage, histological type, histopathologic grade, and residual lesions.

Supplementary Table 4. Subgroup analyses for adjusted hazard ratio (HR) and 95% confidence intervals (CIs) for the association between dietary methionine and betaine intake and total mortality of ovarian cancer (n=635)

Characteristics	Tertiles of energy-adjusted intake *							
	methionine				betaine			
	I	II	III	$P_{interaction}$ **	I	II	III	$P_{interaction}$ **
**Age at diagnosis (years)**				0.33				0.95
≤ 50	1.00 (Ref)	1.25 (0.51-3.05)	1.40 (0.54-3.63)		1.00 (Ref)	1.12 (0.40-3.13)	1.22 (0.46-3.23)	
> 50	1.00 (Ref)	1.36 (0.77-2.42)	0.55 (0.29-1.05)		1.00 (Ref)	1.13 (0.62-2.05)	1.10 (0.62-1.93)	
**Menopausal status**				0.80				0.72
No	1.00 (Ref)	1.72 (0.56-5.27)	1.00 (0.32-3.12)		1.00 (Ref)	0.67 (0.19-2.39)	1.02 (0.32-3.26)	
Yes	1.00 (Ref)	1.38 (0.79-2.40)	0.68 (0.37-1.25)		1.00 (Ref)	1.20 (0.68-2.11)	1.02 (0.58-1.77)	
**Body mass index (kg/m^2)**				0.26				0.16
< 24	1.00 (Ref)	1.35 (0.74-2.48)	0.78 (0.39-1.55)		1.00 (Ref)	1.14 (0.59-2.19)	0.97 (0.50-1.88)	
≥ 24	1.00 (Ref)	0.77 (0.34-1.73)	0.63 (0.27-1.46)		1.00 (Ref)	1.22 (0.54-2.77)	1.31 (0.63-2.76)	
**Alcohol drinking**				0.10				0.91
No	1.00 (Ref)	0.97 (0.57-1.66)	0.63 (0.35-1.14)		1.00 (Ref)	1.14 (0.65-2.01)	1.08 (0.63-1.84)	
Yes	1.00 (Ref)	3.82 (0.96-15.30)	2.36 (0.63-8.80)		1.00 (Ref)	0.63 (0.16-2.55)	0.97 (0.277-3.42)	
**Histological type**				0.64				0.46
Serous	1.00 (Ref)	1.23 (0.70-2.17)	0.87 (0.47-1.60)		1.00 (Ref)	1.09 (0.61-1.97)	1.35 (0.76-2.38)	
Non-serous	1.00 (Ref)	0.68 (0.24-1.95)	0.33 (0.10-1.04)		1.00 (Ref)	1.49 (0.49-4.53)	0.64 (0.23-1.79)	
**FIGO stage**				0.96				0.72
I-II	1.00 (Ref)	1.19 (0.52-2.71)	0.80 (0.28-2.27)		1.00 (Ref)	2.07 (0.80-5.38)	0.89 (0.33-2.36)	

III-IV	1.00 (Ref)	1.06 (0.58-1.94)	0.82 (0.44-1.53)		1.00 (Ref)	0.86 (0.46-1.61)	1.13 (0.64-1.99)	
**Residual lesions**				0.67				0.59
No	1.00 (Ref)	1.06 (0.59-1.89)	0.78 (0.42-1.46)		1.00 (Ref)	1.15 (0.63-2.13)	1.00 (0.55-1.81)	
Yes	1.00 (Ref)	1.94 (0.80-4.67)	1.60 (0.62-4.12)		1.00 (Ref)	1.00 (0.40-2.49)	1.20 (0.52-2.80)	

CI, confidence interval; HR, hazard ratio; Ref, reference.

*Adjusted for energy by the residual method.

** Test for interaction based on strata and dietary methionine and betaine intake.

HR and 95% CI were calculated with the use of the Cox proportional hazards regression model with adjustment for age at diagnosis, body mass index, total energy, alcohol drinking, diet change, education, income, physical activity, menopausal status, parity, multivitamin use, multimineral use, red meat, methyl-donor index, comorbidities, FIGO stage, histological type, histopathologic grade, and residual lesions.

Supplementary Table 5. Adjusted hazard ratio (HR) and 95% confidence interval (CI) of mortality by the tertiles of dietary one-carbon metabolism micronutrients intake among 635 ovarian cancer patients: sensitivity analysis among people who had not taken vitamin supplements *

Variables	Tertiles of energy-adjusted one-carbon metabolism micronutrients intake**			*P* trend †
	I	II	III	
vitamins B_2 (riboflavin) (mg/d)	1.00 (Ref)	0.87 (0.53-1.44)	0.92 (0.57-1.50)	0.65
vitamins B_3 (niacin) (mg/d)	1.00 (Ref)	0.93 (0.59-1.48)	0.75 (0.45-1.26)	0.30
vitamins B_6 (mg/d)	1.00 (Ref)	0.45 (0.28-0.73)	0.50 (0.30-0.81)	< 0.05
vitamins B_9 (folate) (μg/d)	1.00 (Ref)	0.76 (0.47-1.22)	0.77 (0.48-1.24)	0.31
vitamins B_{12} (μg/d)	1.00 (Ref)	1.47 (0.88-2.44)	1.02 (0.62-1.66)	0.72
methionine (mg/d)	1.00 (Ref)	1.27 (0.79-2.05)	0.80 (0.48-1.34)	0.32
choline (mg/d)	1.00 (Ref)	0.63 (0.40-1.01)	0.50 (0.30-0.85)	< 0.05
betaine (mg/d)	1.00 (Ref)	1.10 (0.67-1.80)	1.14 (0.70-1.83)	0.62

CI, confidence interval; HR, hazard ratio; Ref, reference.

* HR and 95% CI were calculated with the use of the Cox proportional hazards regression model with adjustment for age at diagnosis, body mass index, total energy, alcohol drinking, diet change, education, income, physical activity, menopausal status, parity, multivitamin use, multimineral use, red meat, methyl-donor index, comorbidities, FIGO stage, histological type, histopathologic grade, and residual lesions.

** Energy adjustment by residual method.

† Test for trend based on variables containing the median value for each tertile.

Supplementary Table 6. Adjusted hazard ratio (HR) and 95% confidence interval (CI) of mortality by the tertiles of dietary one-carbon metabolism micronutrients intake among 635 ovarian cancer patients: sensitivity analysis by mutually adjusted for all of the dietary one-carbon metabolism micronutrients *

Variables	Tertiles of energy-adjusted one-carbon metabolism micronutrients intake**			*P* trend †
	I	II	III	
vitamins B_2 (riboflavin) (mg/d)	1.00 (ref)	1.41 (0.81-2.45)	2.07 (1.02-4.22)	< 0.05
vitamins B_3 (niacin) (mg/d)	1.00 (ref)	1.04 (0.65-1.68)	0.67 (0.40-1.11)	0.16
vitamins B_6 (mg/d)	1.00 (ref)	0.45 (0.27-0.74)	0.48 (0.25-0.92)	< 0.05
vitamins B_9 (folate) (μg/d)	1.00 (ref)	0.91 (0.56-1.48)	1.02 (0.56-1.85)	0.95
vitamins B_{12} (μg/d)	1.00 (ref)	1.36 (0.82-2.26)	1.10 (0.67-1.82)	0.94
methionine (mg/d)	1.00 (ref)	1.29 (0.80-2.08)	0.90 (0.52-1.54)	0.63
choline (mg/d)	1.00 (ref)	0.57 (0.34-0.96)	0.31 (0.15-0.62)	< 0.05
betaine (mg/d)	1.00 (ref)	1.40 (0.83-2.33)	1.67 (0.98-2.86)	0.07

CI, confidence interval; HR, hazard ratio; Ref, reference.

* HR and 95% CI were calculated with the use of the Cox proportional hazards regression model with adjustment for age at diagnosis, body mass index, total energy, alcohol drinking, diet change, education, income, physical activity, menopausal status, parity, multivitamin use, multimineral use, red meat, methyl-donor index, comorbidities, FIGO stage, histological type, histopathologic grade, residual lesions, and all of the dietary one-carbon metabolism micronutrients.

** Energy adjustment by residual method.

† Test for trend based on variables containing the median value for each tertile.

Suppplementary Table 7. Adjusted hazard ratio (HR) and 95% confidence intervals (CIs) for the association between dietary one-carbon metabolism micronutrients intake and total mortality of ovarian cancer (n=635) *

Characteristics	Quartiles of energy-adjusted intake **				*P* trend †	Continuous ‡
	I	II	III	IV		
**Methionine (Range, mg/d)**	< 951.08	951.08-1032.06	1032.06-1147.33	≥ 1147.33		
Deaths, N (% of total deaths)	31 (27.19)	25 (21.93)	30 (26.32)	28 (24.56)		
Model 1	1.00 (Ref)	0.71 (0.42-1.20)	0.87 (0.52-1.44)	0.75 (0.45-1.25)	0.41	0.92 (0.79-1.06)
Model 2	1.00 (Ref)	0.70 (0.40-1.22)	0.88 (0.51-1.50)	0.82 (0.48-1.41)	0.35	0.93 (0.80-1.07)
Model 3	1.00 (Ref)	0.67 (0.38-1.17)	0.87 (0.50-1.51)	0.80 (0.46-1.39)	0.67	0.93 (0.80-1.07)
**Vitamins B_2 (riboflavin) (Range, mg/d)**	< 0.78	0.78-0.88	0.88-1.01	≥ 1.01		
Deaths, N (% of total deaths)	34 (29.82)	25 (21.93)	29 (25.44)	26 (22.81)		
Model 1	1.00 (Ref)	0.70 (0.42-1.18)	0.82 (0.50-1.35)	0.73 (0.44-1.22)	0.31	0.90 (0.73-1.11)
Model 2	1.00 (Ref)	0.67 (0.39-1.14)	0.79 (0.47-1.32)	0.74 (0.44-1.26)	0.35	0.91 (0.74-1.11)
Model 3	1.00 (Ref)	0.78 (0.45-1.34)	0.87 (0.51-1.47)	0.79 (0.46-1.36)	0.39	0.93 (0.76-1.14)
**Vitamins B_3 (niacin) (Range, mg/d)**	< 11.81	11.81-13.86	13.86-15.27	≥ 15.27		
Deaths, N (% of total deaths)	32 (28.07)	29 (25.44)	29 (25.44)	24 (21.05)		
Model 1	1.00 (Ref)	0.94 (0.57-1.56)	0.87 (0.53-1.45)	0.69 (0.41-1.19)	0.19	0.89 (0.69-1.15)

Model 2	1.00 (Ref)	0.97 (0.57-1.65)	0.87 (0.51-1.49)	0.66 (0.37-1.19)	0.17	0.88 (0.67-1.17)
Model 3	1.00 (Ref)	1.09 (0.64-1.86)	0.88 (0.51-1.53)	0.76 (0.42-1.38)	0.32	0.93 (0.70-1.24)
**Vitamins B_6 (Range, mg/d)**	< 0.37	0.37-0.43	0.43-0.52	≥ 0.52		
Deaths, N (% of total deaths)	35 (30.70)	33 (28.95)	22 (19.30)	24 (21.05)		
Model 1	1.00 (Ref)	0.79 (0.49-1.27)	0.53 (0.31-0.91)	0.60 (0.35-1.00)	< 0.05	0.75 (0.58-0.97)
Model 2	1.00 (Ref)	0.69 (0.42-1.15)	0.43 (0.24-0.76)	0.54 (0.31-0.93)	< 0.05	0.73 (0.57-0.95)
Model 3	1.00 (Ref)	0.63 (0.37-1.06)	0.39 (0.22-0.70)	0.50 (0.29-0.89)	< 0.05	0.70 (0.53-0.92)
**Vitamins B_9 (folate) (Range, µg/d)**	< 170.13	170.13-207.67	207.67-250.07	≥ 250.07		
Deaths, N (% of total deaths)	31 (27.19)	27 (23.68)	27 (23.68)	29 (25.45)		
Model 1	1.00 (Ref)	0.80 (0.48-1.33)	0.85 (0.51-1.42)	0.82 (0.49-1.36)	0.52	0.85 (0.70-1.04)
Model 2	1.00 (Ref)	0.68 (0.40-1.17)	0.73 (0.43-1.24)	0.75 (0.44-1.25)	0.37	0.82 (0.67-1.00)
Model 3	1.00 (Ref)	0.61 (0.35-1.04)	0.60 (0.45-1.04)	0.77 (0.46-1.31)	0.47	0.81 (0.65-1.00)
**Vitamins B_{12} (Range, µg/d)**	< 0.03	0.03-0.08	0.08-0.17	≥ 0.17		
Deaths, N (% of total deaths)	26 (22.81)	26 (22.81)	35 (30.70)	27 (23.68)		
Model 1	1.00 (Ref)	0.96 (0.56-1.65)	1.37 (0.82-2.28)	0.95 (0.55-1.63)	0.85	1.02 (0.91-1.16)
Model 2	1.00 (Ref)	1.17 (0.64-2.12)	1.55 (0.88-2.73)	0.99 (0.56-1.73)	0.70	1.00 (0.89-1.14)
Model 3	1.00 (Ref)	1.21 (0.66-2.23)	1.48 (0.82-12.65)	0.99 (0.56-1.74)	0.66	1.02 (0.90-1.17)

**Choline (Range, mg/d)**	< 232.28	232.28-275.04	275.04-325.91	≥ 325.91		
Deaths, N (% of total deaths)	40 (35.09)	25 (21.93)	26 (22.81)	23 (20.17)		
Model 1	1.00 (Ref)	0.56 (0.34-0.92)	0.61 (0.37-1.00)	0.52 (0.31-0.86)	< 0.05	0.78 (0.61-0.98)
Model 2	1.00 (Ref)	0.49 (0.29-0.82)	0.53 (0.32-0.88)	0.52 (0.30-0.90)	< 0.05	0.76 (0.60-0.97)
Model 3	1.00 (Ref)	0.46 (0.27-0.79)	0.55 (0.33-0.93)	0.51 (0.29-0.90)	< 0.05	0.79 (0.61-1.01)
**Betaine (Range, mg/d)**	< 35.95	35.95-49.22	49.22-72.18	≥ 72.18		
Deaths, N (% of total deaths)	26 (22.81)	31 (27.19)	28 (24.56)	29 (25.44)		
Model 1	1.00 (Ref)	1.12 (0.66-1.89)	1.03 (0.60-1.74)	1.04 (0.61-1.77)	0.99	1.01 (0.86-1.20)
Model 2	1.00 (Ref)	1.21 (0.69-2.12)	1.09 (0.62-1.91)	1.12 (0.65-1.93)	0.82	1.01 (0.87-1.20)
Model 3	1.00 (Ref)	1.16 (0.66-2.04)	1.07 (0.61-1.88)	1.00 (0.58-1.93)	0.87	0.98 (0.83-1.16)

CI, confidence interval; HR, hazard ratio; Ref, reference.

* HR and 95% CI were calculated with the use of the Cox proportional hazards regression model.

** Adjusted for energy by the residual method.

† Test for trend based on variables containing the median value for each quartile.

‡ Continuous intakes were calculated by per unit increase.

Model 1 adjusted for age at diagnosis and body mass index.

Model 2 adjusted for age at diagnosis, total energy, body mass index, alcohol drinking, diet change, education, income, physical activity, menopausal status, parity,

multivitamin use, multimineral use, red meat, and methyl-donor index.

Model 3 adjusted for age at diagnosis, total energy, body mass index, alcohol drinking, diet change, education, income, physical activity, menopausal status, parity, multivitamin use, multimineral use, red meat, methyl-donor index, comorbidities, FIGO stage, histological type, histopathologic grade, and residual lesions.

Supplementary Table 8. Adjusted hazard ratio (HR) and 95% confidence intervals (CIs) for the association between dietary one-carbon metabolism micronutrients intake and total mortality of ovarian cancer (n=635) *

Micronutrients **	No. of patients/deaths	HR (95% CI) *		
		Model 1	Model 2	Model 3
**Vitamins B_2 (riboflavin) (mg/d)**				
< 1.2	599/107	1.00 (Ref)	1.00 (Ref)	1.00 (Ref)
≥ 1.2	36/7	1.08 (0.50-2.33)	0.98 (0.44-2.18)	1.05 (0.47-2.34)
**Vitamins B_3 (niacin) (mg/d)**				
< 12	169/33	1.00 (Ref)	1.00 (Ref)	1.00 (Ref)
≥ 12	466/81	0.85 (0.57-1.28)	0.89 (0.58-1.38)	0.95 (0.61-1.46)
**Vitamins B_9 (folate) (µg/d)**				
< 400	624/113	1.00 (Ref)	1.00 (Ref)	1.00 (Ref)
≥ 400	11/1	0.54 (0.08-3.87)	0.38 (0.05-2.50)	0.35 (0.05-2.68)
**Choline (mg/d)**				
< 400	606/109	1.00 (Ref)	1.00 (Ref)	1.00 (Ref)
≥ 400	29/5	0.85 (0.35-2.08)	0.74 (0.29-1.87)	0.73 (0.29-1.83)

CI, confidence interval; HR, hazard ratio; Ref, reference.

* HR and 95% CI were calculated with the use of the Cox proportional hazards regression model.

** Adjusted for energy by the residual method.

Model 1 adjusted for age at diagnosis and body mass index.

Model 2 adjusted for age at diagnosis, total energy, body mass index, alcohol drinking, diet change, education, income, physical activity, menopausal status, parity, multivitamin use, multimineral use, red meat, and methyl-donor index.

Model 3 adjusted for age at diagnosis, total energy, body mass index, alcohol drinking, diet change, education, income, physical activity, menopausal status, parity, multivitamin use, multimineral use, red meat, methyl-donor index, comorbidities, FIGO stage, histological type, histopathologic grade, and residual lesions.

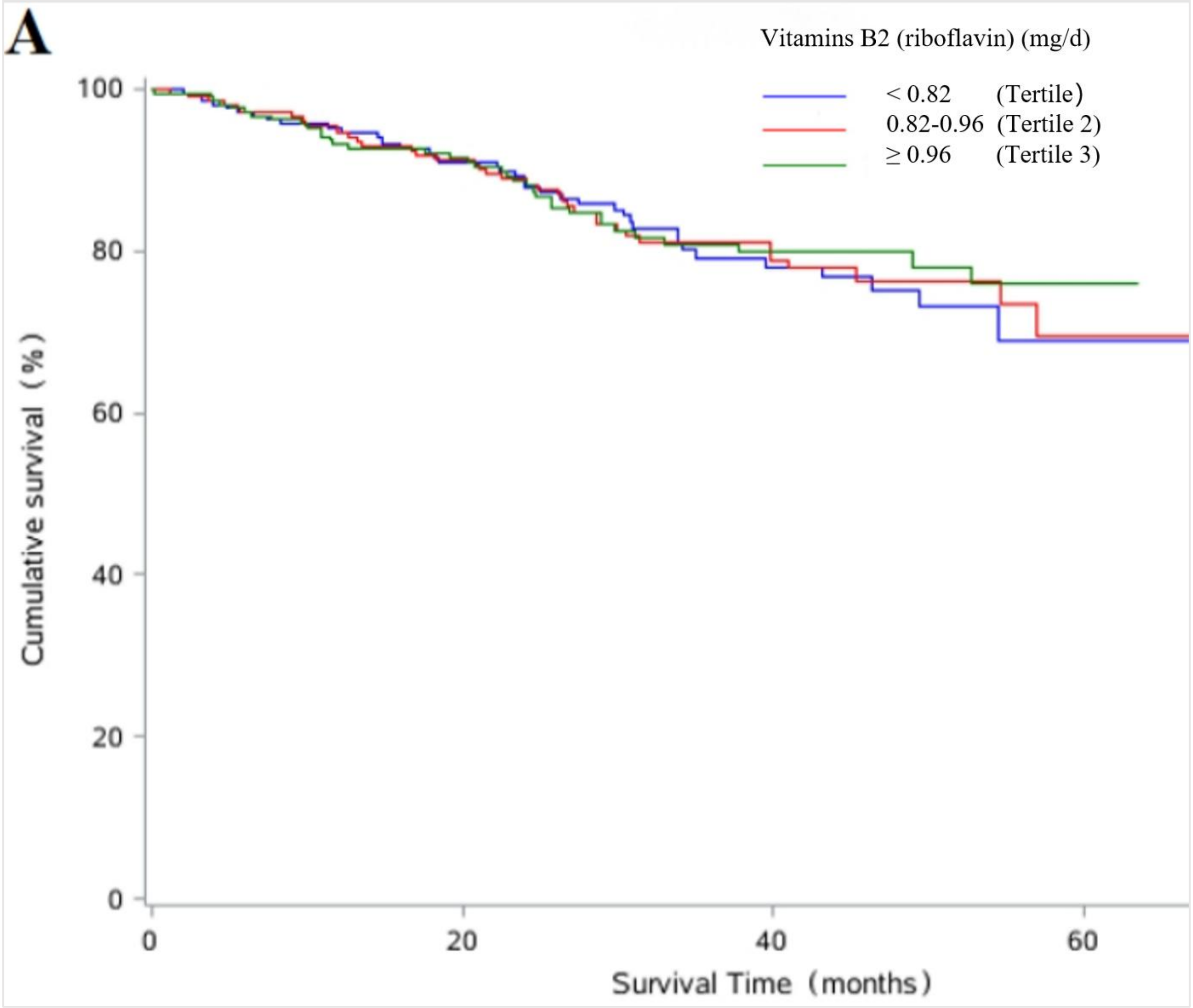

A
Vitamins B2 (riboflavin) (mg/d)
< 0.82 (Tertile)
0.82-0.96 (Tertile 2)
≥ 0.96 (Tertile 3)
100
80
60
40
20
0
Cumulative survival（%）
0
20
40
60
Survival Time（months）

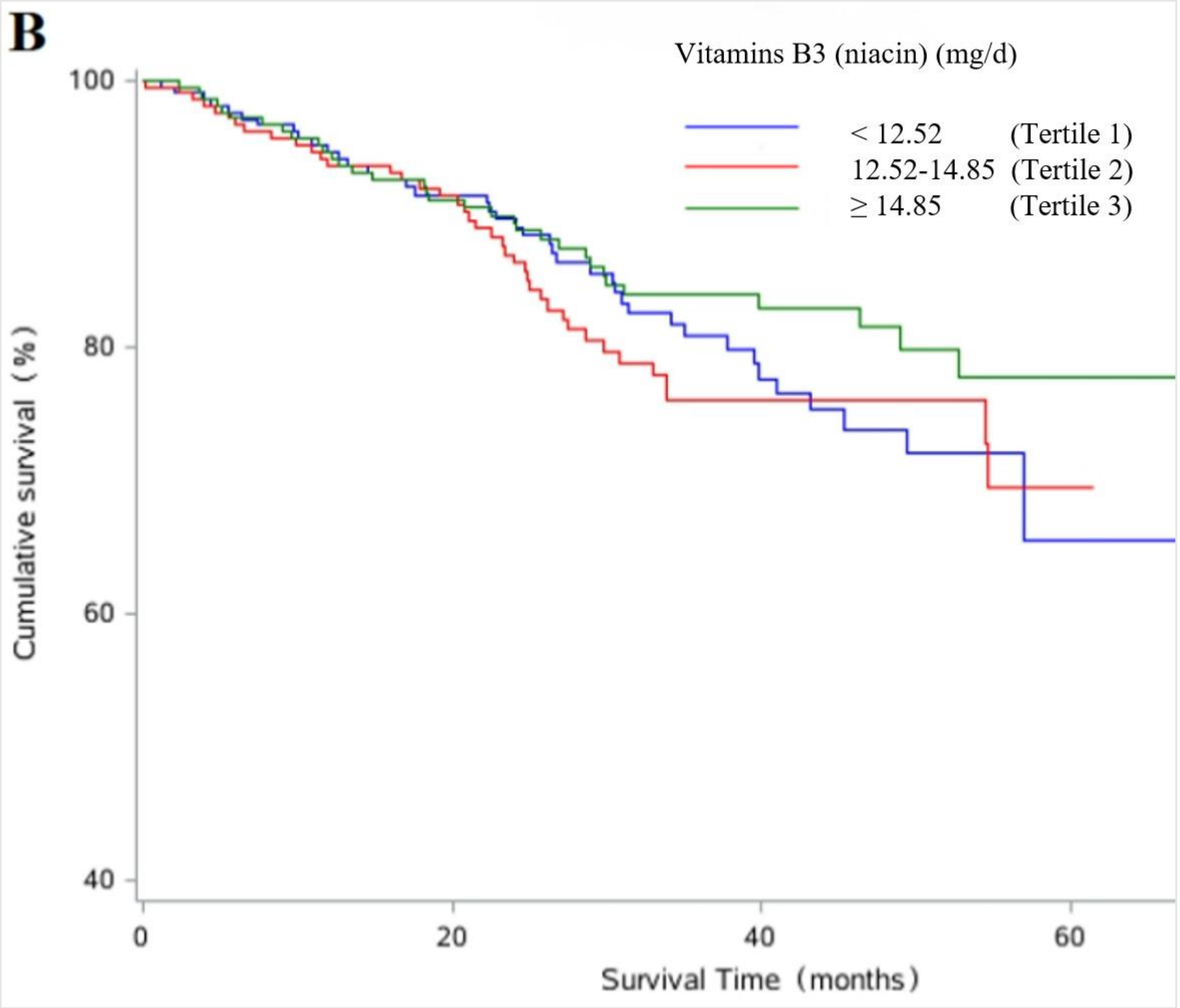

B
Vitamins B3 (niacin) (mg/d)
< 12.52 (Tertile 1)
12.52-14.85 (Tertile 2)
≥ 14.85 (Tertile 3)
100
80
60
40
Cumulative survival（%）
0
20
40
60
Survival Time（months）

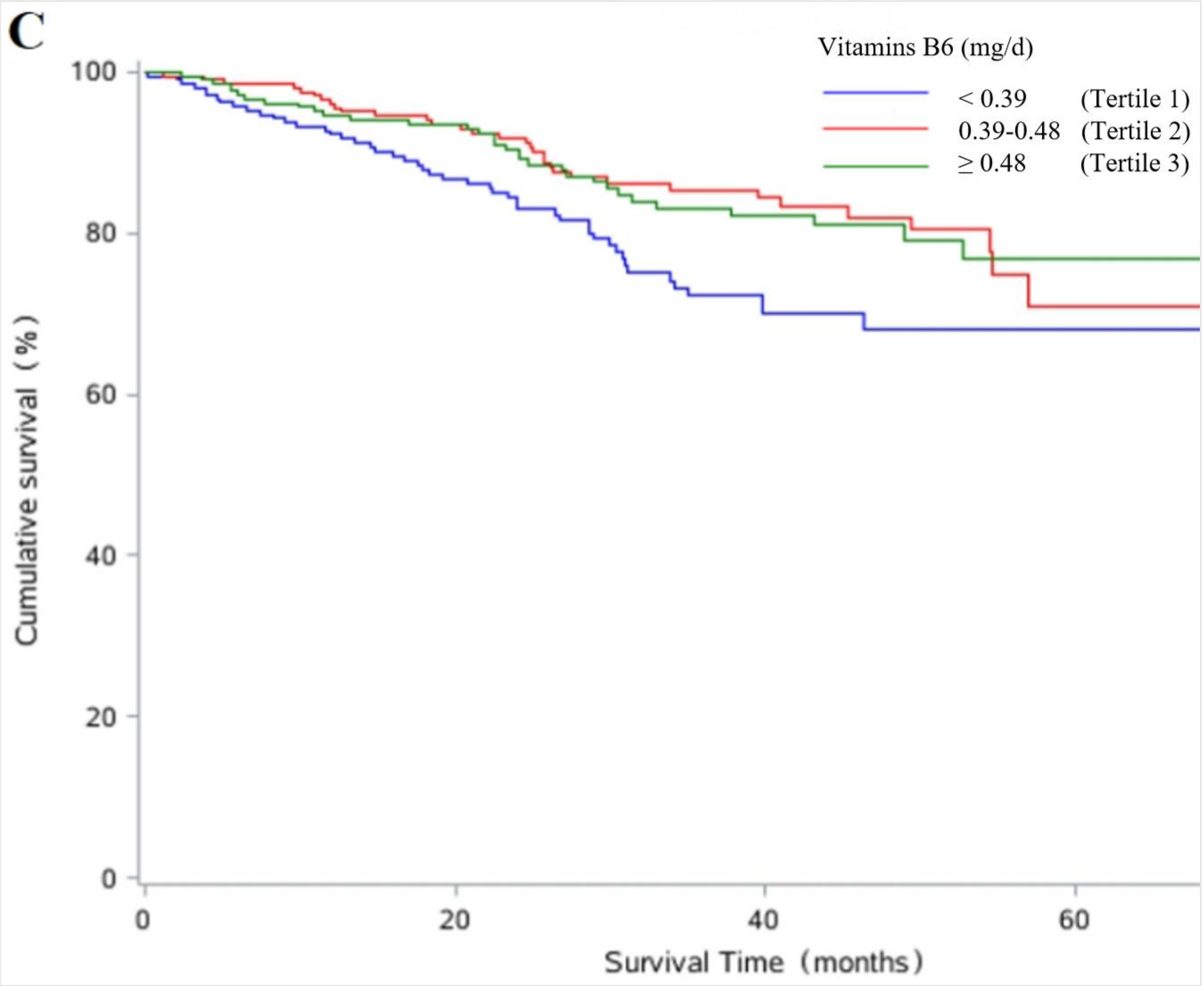
C
Vitamins B6 (mg/d)
< 0.39 (Tertile 1)
0.39-0.48 (Tertile 2)
≥ 0.48 (Tertile 3)
Cumulative survival（%）
100
80
60
40
20
0
0
20
40
60
Survival Time（months）

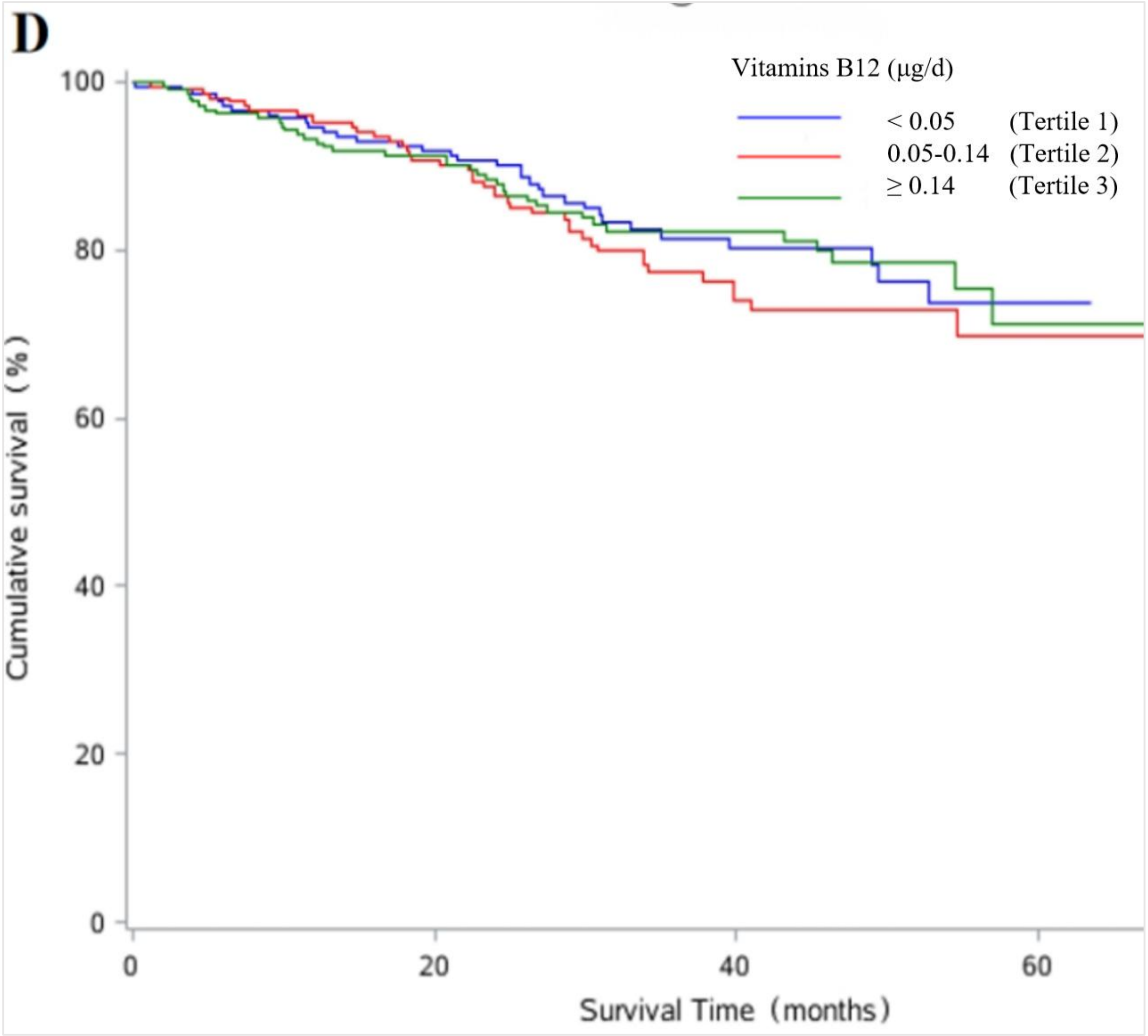
D
Vitamins B12 (μg/d)
< 0.05 (Tertile 1)
0.05-0.14 (Tertile 2)
≥ 0.14 (Tertile 3)
Cumulative survival（%）
Survival Time（months）
0
20
40
60
80
100

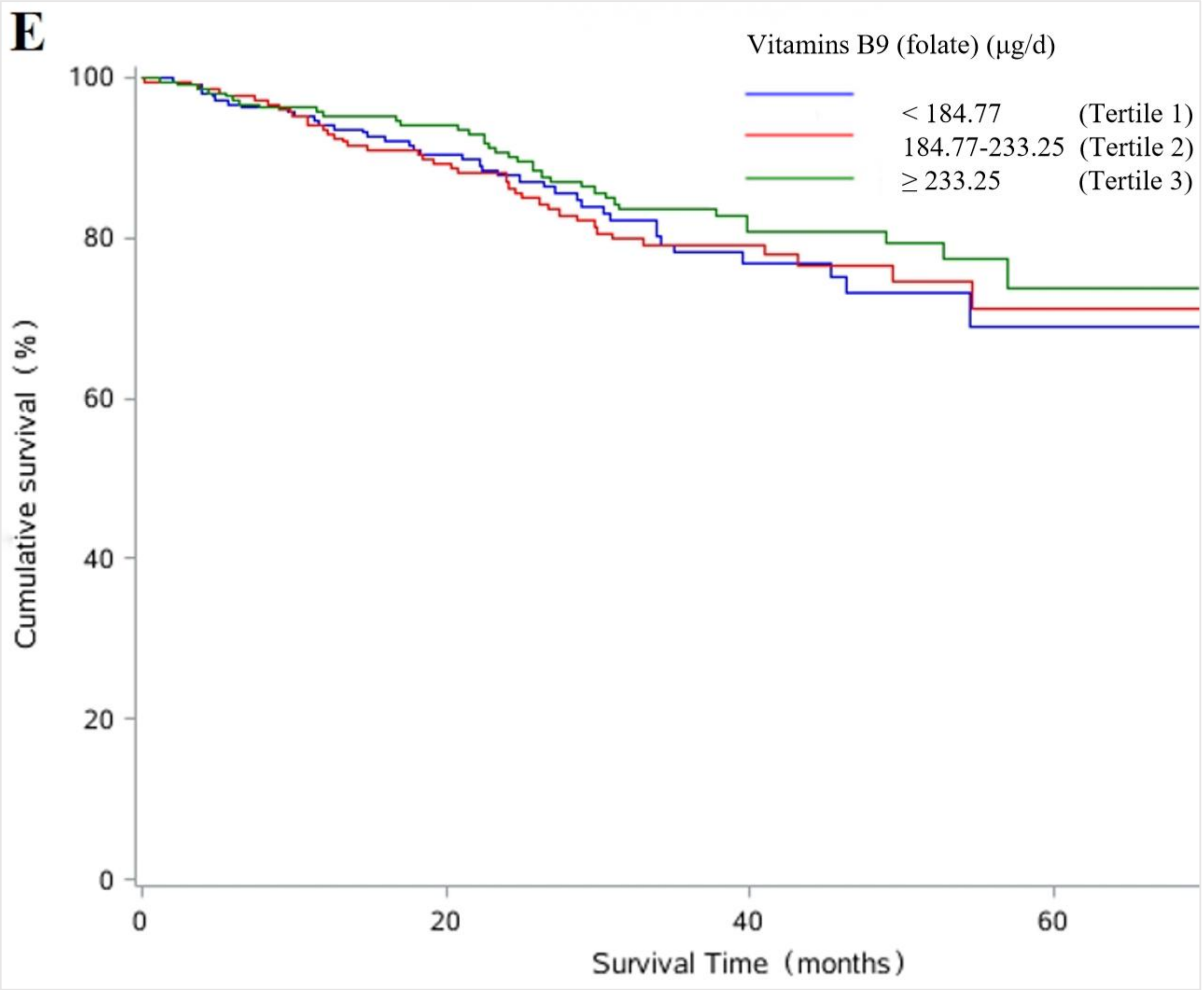
E
Vitamins B9 (folate) (μg/d)
< 184.77 (Tertile 1)
184.77-233.25 (Tertile 2)
≥ 233.25 (Tertile 3)
Cumulative survival（%）
100
80
60
40
20
0
0
20
40
60
Survival Time（months）

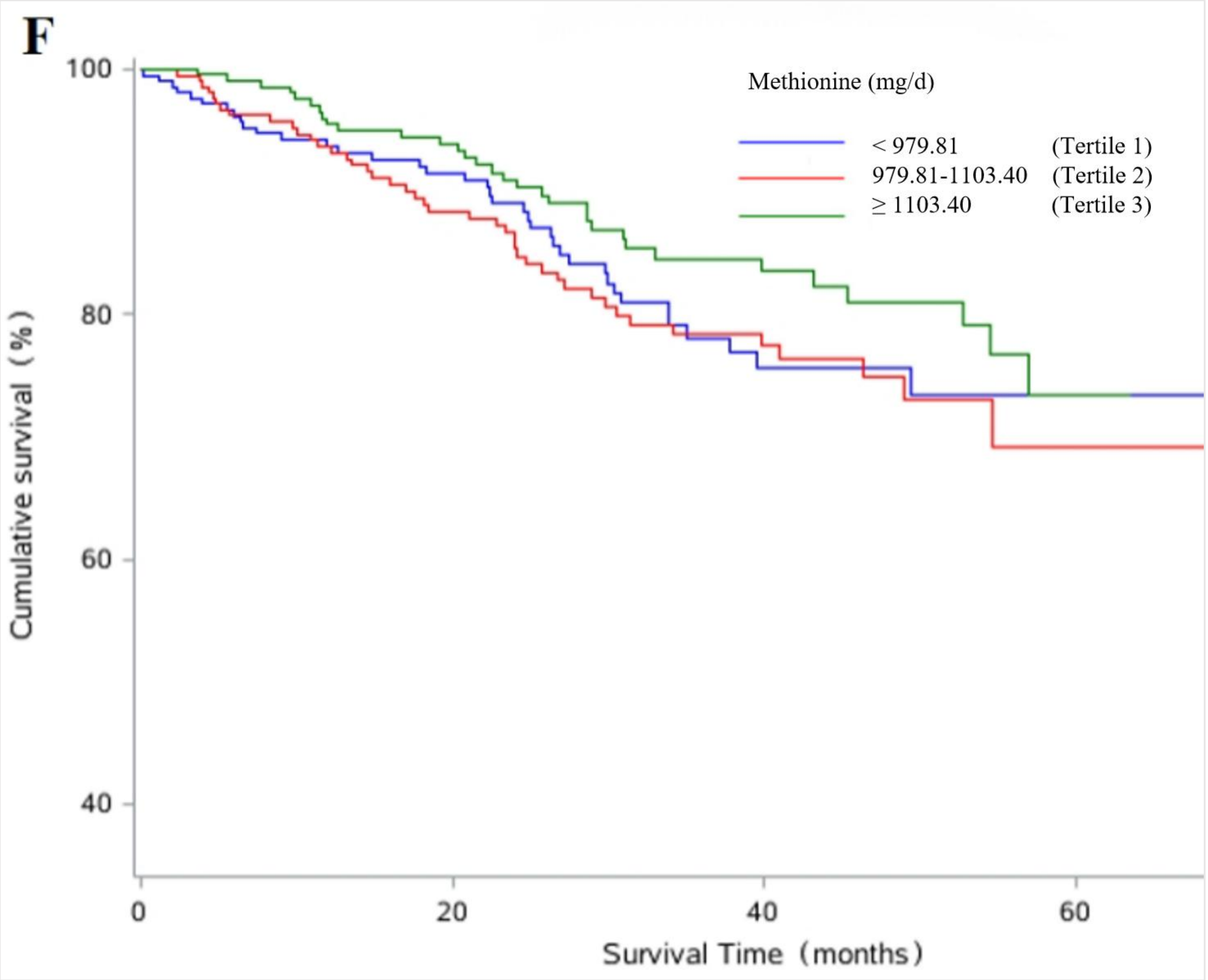
F
Methionine (mg/d)
< 979.81 (Tertile 1)
979.81-1103.40 (Tertile 2)
≥ 1103.40 (Tertile 3)
Cumulative survival（%）
100
80
60
40
0
20
40
60
Survival Time（months）

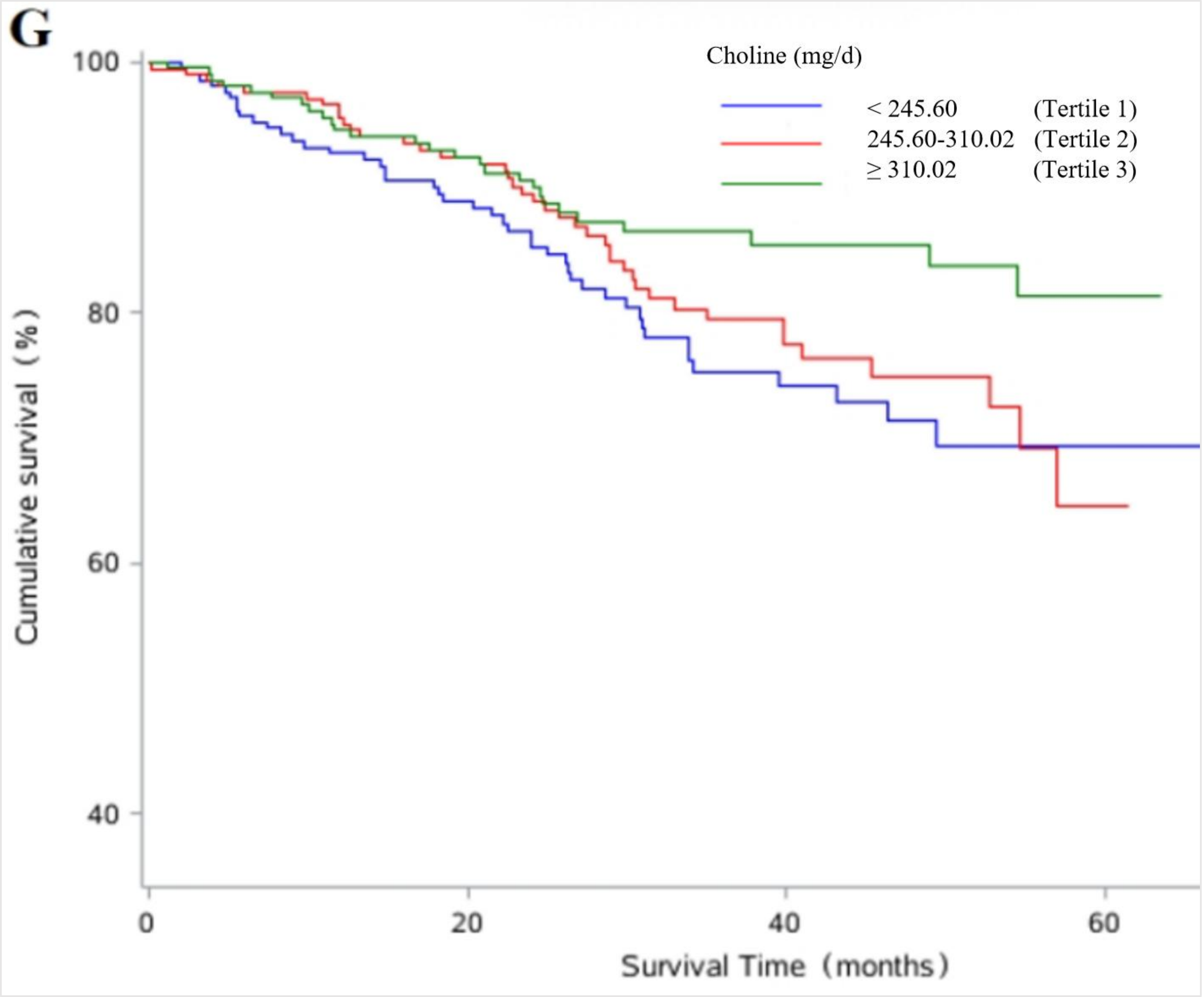
G
Choline (mg/d)
< 245.60 (Tertile 1)
245.60-310.02 (Tertile 2)
≥ 310.02 (Tertile 3)
100
80
60
40
Cumulative survival（%）
0
20
40
60
Survival Time（months）

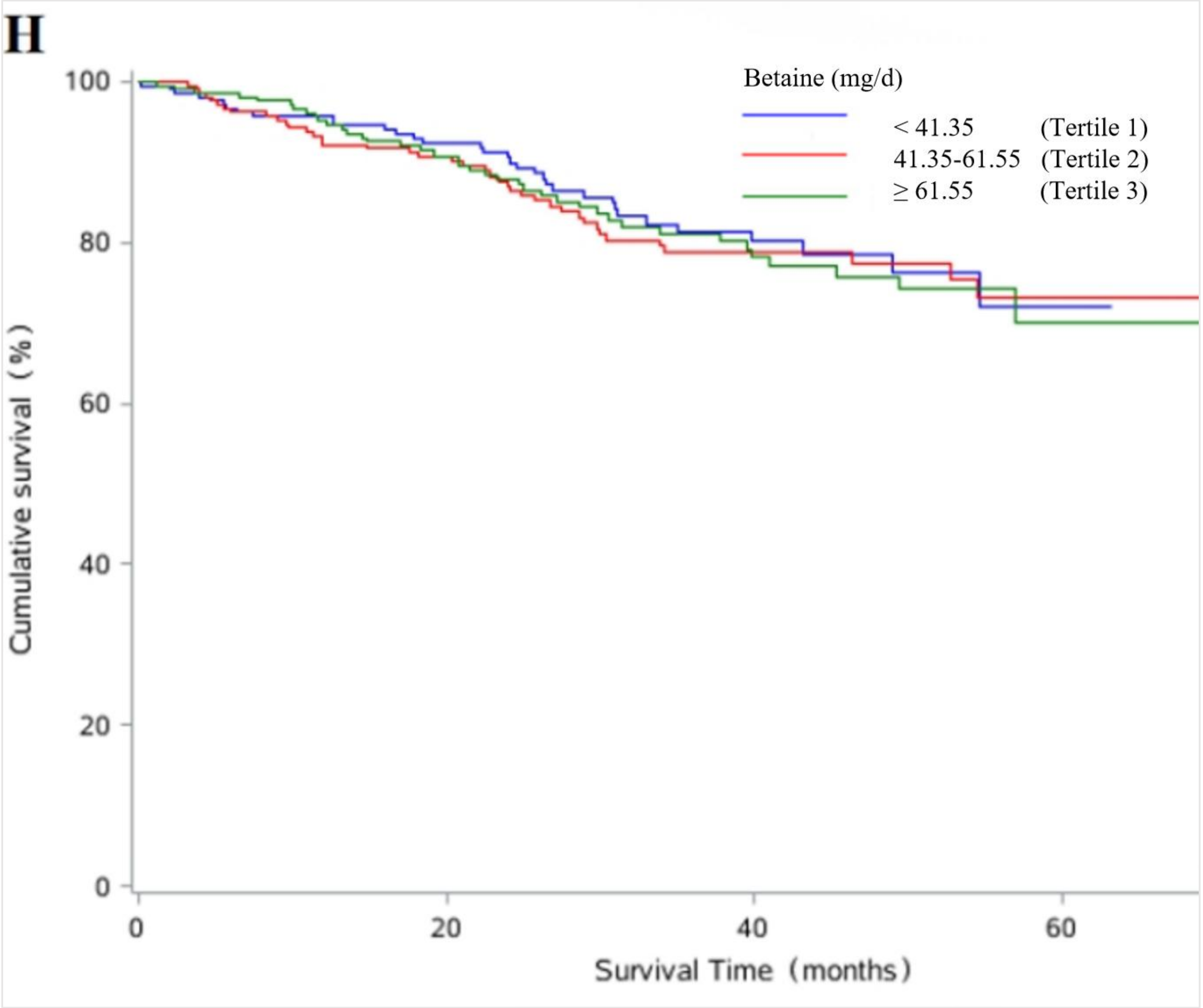

H
Betaine (mg/d)
< 41.35 (Tertile 1)
41.35-61.55 (Tertile 2)
≥ 61.55 (Tertile 3)
Cumulative survival（%）
Survival Time（months）
100
80
60
40
20
0
0
20
40
60

Supplementary Figure 1. Kaplan–Meier survival curves for vitamins B2 (A), vitamins B3 (B), vitamins B6 (C), vitamins B12 (D), folate (E), methionine (F), choline (G), and betaine (H).

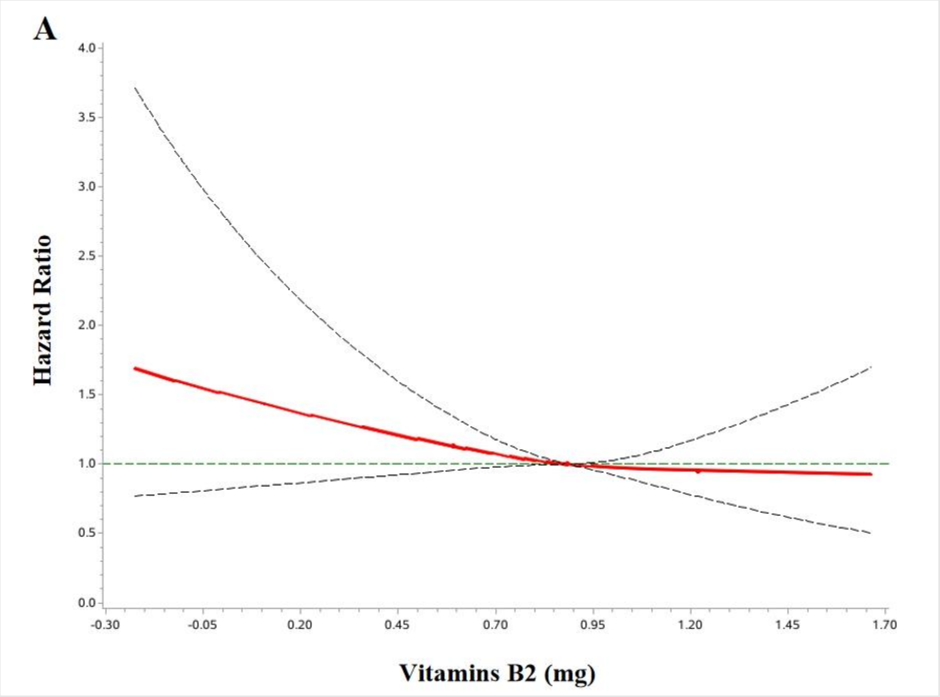
A
Hazard Ratio
4.0
3.5
3.0
2.5
2.0
1.5
1.0
0.5
0.0
-0.30
-0.05
0.20
0.45
0.70
0.95
1.20
1.45
1.70
Vitamins B2 (mg)

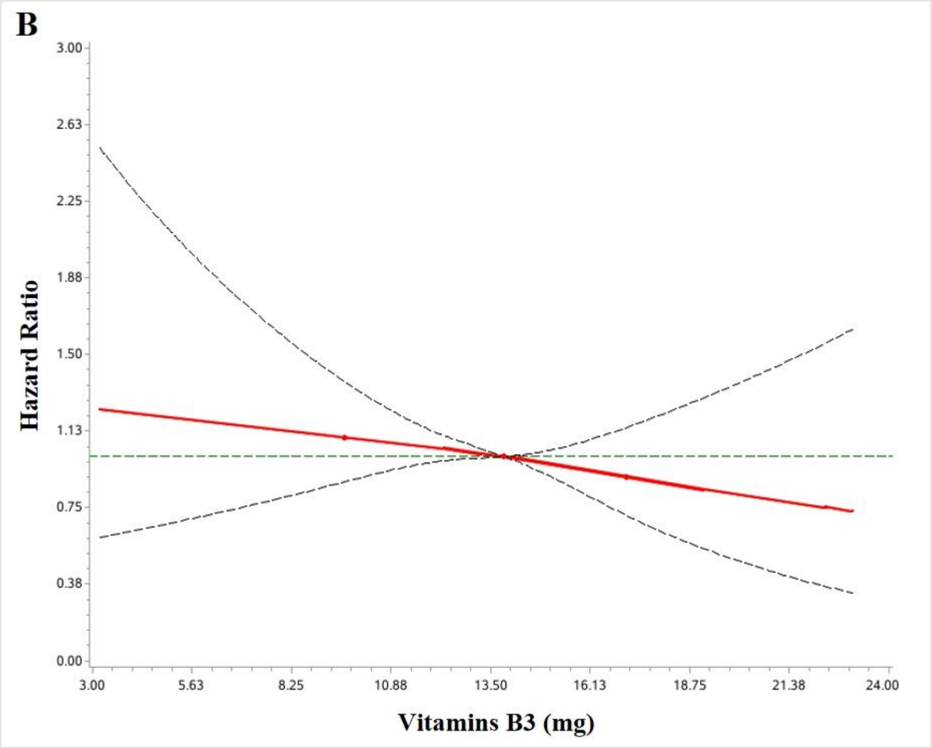
B
Hazard Ratio
3.00
2.63
2.25
1.88
1.50
1.13
0.75
0.38
0.00
3.00
5.63
8.25
10.88
13.50
16.13
18.75
21.38
24.00
Vitamins B3 (mg)

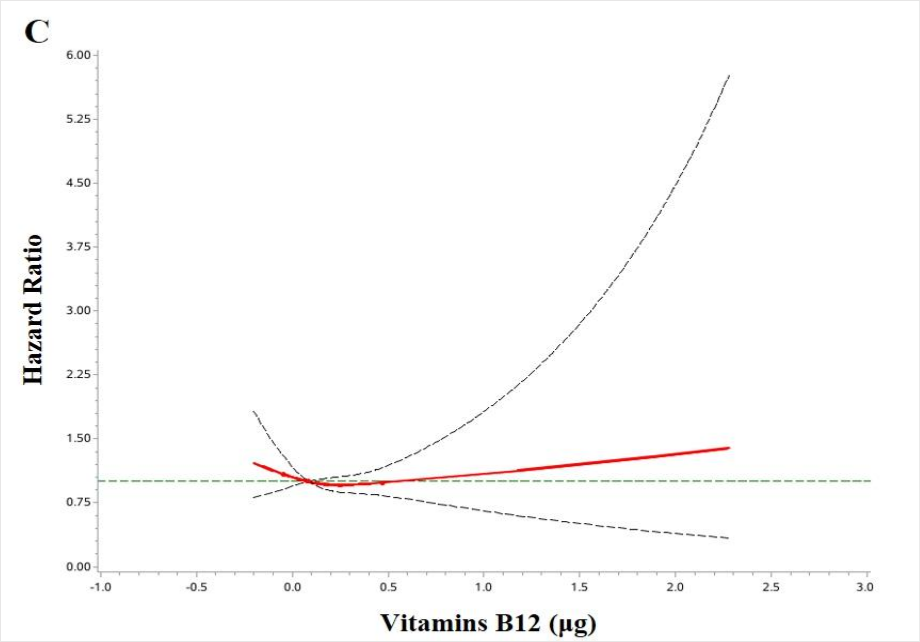
C
Hazard Ratio
6.00
5.25
4.50
3.75
3.00
2.25
1.50
0.75
0.00
-1.0
-0.5
0.0
0.5
1.0
1.5
2.0
2.5
3.0
Vitamins B12 (μg)

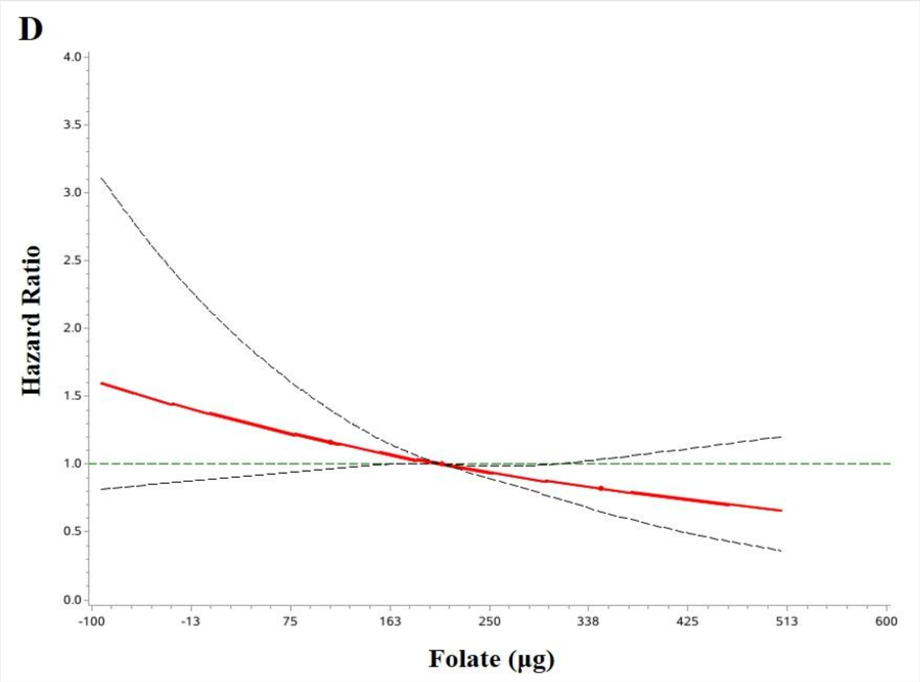
D
Hazard Ratio
4.0
3.5
3.0
2.5
2.0
1.5
1.0
0.5
0.0
-100
-13
75
163
250
338
425
513
600
Folate (μg)

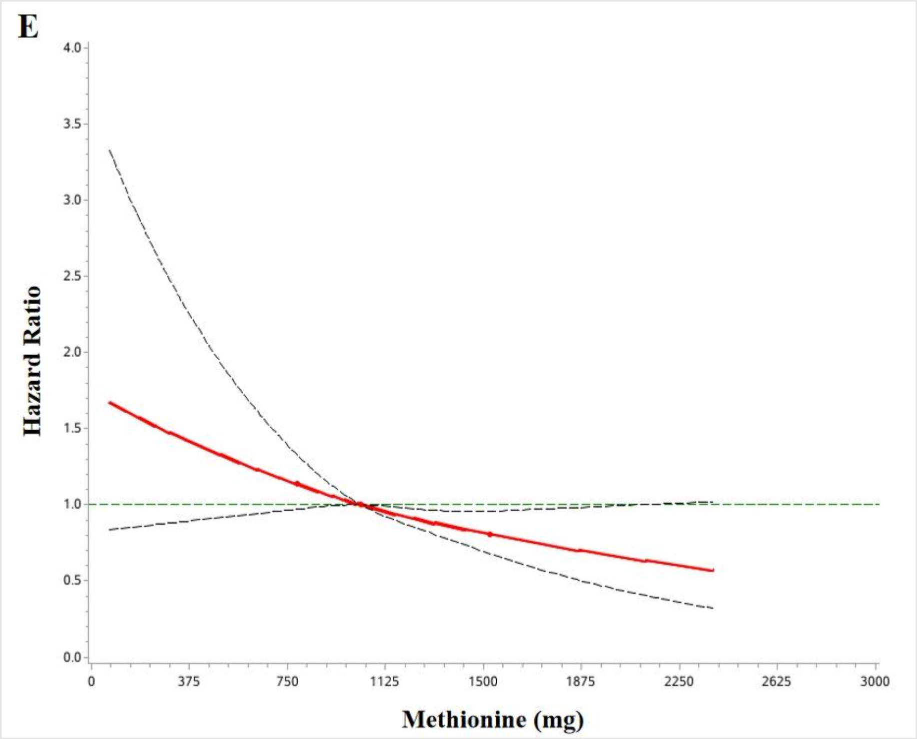

E
Hazard Ratio
4.0
3.5
3.0
2.5
2.0
1.5
1.0
0.5
0.0
0
375
750
1125
1500
1875
2250
2625
3000
Methionine (mg)

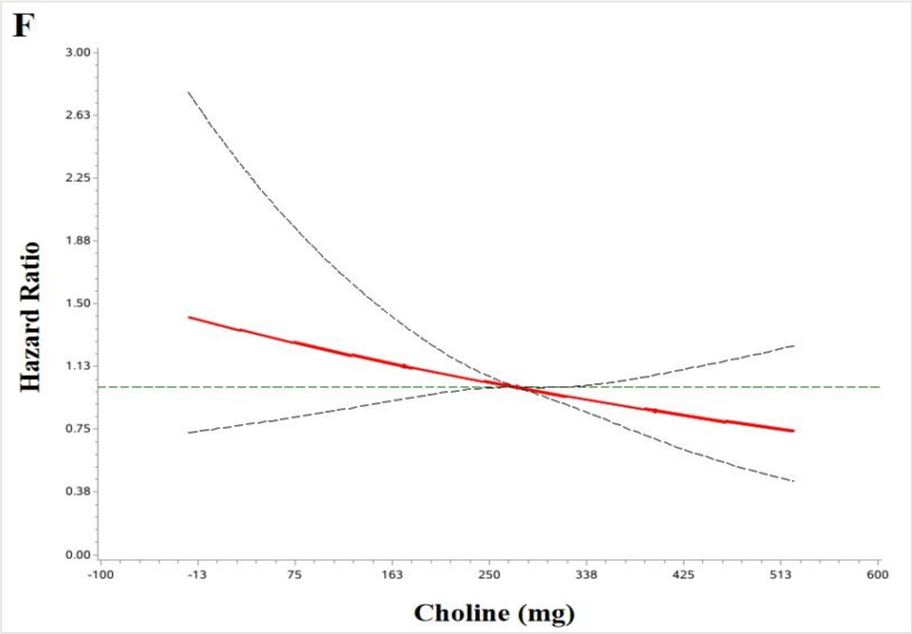
F
Hazard Ratio
3.00
2.63
2.25
1.88
1.50
1.13
0.75
0.38
0.00
-100
-13
75
163
250
338
425
513
600
Choline (mg)

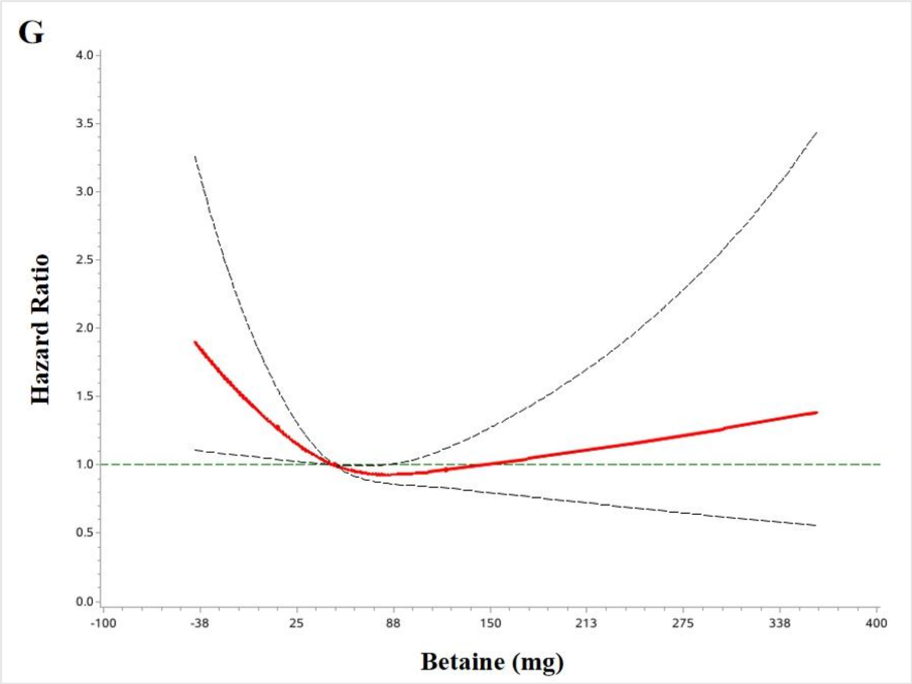

Supplementary Figure 2. HR (and 95% CI) of overall survival among OC patients by vitamins B2 (A), vitamins B3 (B), vitamins B12 (C), folate (D), methionine (E), choline (F), and betaine (G). The association was adjusted for age at diagnosis, body mass index, total energy, alcohol drinking, diet change, education, income, physical activity, menopausal status, parity, multivitamin use, multimineral use, red meat, methyl-donor index, comorbidities, FIGO stage, histological type, histopathologic grade, and residual lesions.
